# Biosynthesis and Characterization of Zearalenone-14-Sulfate, Zearalenone-14-Glucoside and Zearalenone-16-Glucoside Using Common Fungal Strains

**DOI:** 10.3390/toxins10030104

**Published:** 2018-03-01

**Authors:** Antje Borzekowski, Tatjana Drewitz, Julia Keller, Dietmar Pfeifer, Hans-Jörg Kunte, Matthias Koch, Sascha Rohn, Ronald Maul

**Affiliations:** 1 Department Analytical Chemistry, Reference Materials, Bundesanstalt für Materialforschung und-prüfung (BAM), Richard-Willstätter-Str. 11, 12489 Berlin, Germany; antje.borzekowski@bam.de (A.B.); tatjana.drewitz@bam.de (T.D.); julia.keller@bam.de (J.K.); dietmar.pfeifer@bam.de (D.P.); 2 Department Materials and the Environment, Bundesanstalt für Materialforschung und-prüfung (BAM), Unter den Eichen 87, 12205 Berlin, Germany; hans-joerg.kunte@bam.de; 3 Institute of Food Chemistry, Hamburg School of Food Science, University of Hamburg, Grindelallee 117, 20146 Hamburg, Germany; rohn@chemie.uni-hamburg.de (S.R.); ronald.maul@chemie.uni-hamburg.de (R.M.); 4 German Federal Institute for Risk Assessment (BfR), Max-Dohrn-Str. 8-10, 10589 Berlin, Germany

**Keywords:** mycotoxin, zearalenone, conjugate, biosynthesis, *Fusarium*, *Aspergillus*, *Rhizopus*

## Abstract

Zearalenone (ZEN) and its phase II sulfate and glucoside metabolites have been detected in food and feed commodities. After consumption, the conjugates can be hydrolyzed by the human intestinal microbiota leading to liberation of ZEN that implies an underestimation of the true ZEN exposure. To include ZEN conjugates in routine analysis, reliable standards are needed, which are currently not available. Thus, the aim of the present study was to develop a facilitated biosynthesis of ZEN-14-sulfate, ZEN-14-glucoside and ZEN-16-glucoside. A metabolite screening was conducted by adding ZEN to liquid fungi cultures of known ZEN conjugating *Aspergillus* and *Rhizopus* strains. Cultivation conditions and ZEN incubation time were varied. All media samples were analyzed for metabolite formation by HPLC-MS/MS. In addition, a consecutive biosynthesis was developed by using *Fusarium graminearum* for ZEN biosynthesis with subsequent conjugation of the toxin by utilizing *Aspergillus* and *Rhizopus* species. ZEN-14-sulfate (yield: 49%) is exclusively formed by *Aspergillus oryzae*. ZEN-14-glucoside (yield: 67%) and ZEN-16-glucoside (yield: 39%) are formed by *Rhizopus oryzae* and *Rhizopus* *oligosporus*, respectively. Purities of ≥73% ZEN-14-sulfate, ≥82% ZEN-14-glucoside and ≥50% ZEN-16-glucoside were obtained by ^1^H-NMR. In total, under optimized cultivation conditions, fungi can be easily utilized for a targeted and regioselective synthesis of ZEN conjugates.

## 1. Introduction

Zearalenone (ZEN) is a mycotoxin produced by *Fusarium* species, including *Fusarium graminearum* [1,2]. *Fusarium* spp. infest for instance wheat, maize and barley. Therefore, ZEN is a common contaminant in cereal-based feed and food products [3,4]. For many commodities, legal limits for ZEN have been established in various countries worldwide. In the metabolism of the infested plant and in fungal metabolism, sulfate and glucoside conjugates of ZEN are formed. Kovalsky-Paris et al. [5] reported the conversion of ZEN to ZEN-14-glucoside (ZEN-14-G) and ZEN-16-glucoside (ZEN-16-G) in barley. Krenn et al. [6] described the production of ZEN-14-G by the model plant *Arabidopsis thaliana* after ZEN addition. ZEN-14-sulfate (ZEN-14-S) was found to be a natural fungal metabolite of *Fusarium*, *Rhizopus*, and *Aspergillus* species [2,7,8]. *Rhizopus oryzae*, *Aspergillus oryzae,* and *Aspergillus niger* in particular convert ZEN very rapidly to ZEN-14-S. A study from De Boevre et al. [3] on the occurrence of ZEN and its metabolites showed that food and feed products can be highly contaminated with these compounds. One hundred and seventy-four cereal-based food products and 67 compound feeds were analyzed for the occurrence of ZEN, the phase I metabolite zearalenol (ZEL) and the glucoside conjugates of ZEN and ZEL, and ZEN sulfate. For example, the analyzed cornflakes contained ZEN-14-G and ZEN-14-S with average levels of 39 µg/kg and 23 µg/kg and maximum levels of 369 µg/kg and 417 µg/kg, respectively. Also, the sum of the ZEN metabolites in cornflakes (144 µg/kg, mean value) exceeded the amount of the parent compound (76 µg/kg, mean value).

ZEN is regulated by the European Union (EU) with maximum levels from 20 to 400 µg/kg for cereals and cereal products [9], because of its estrogenic activity. ZEN is a mycroestrogen and can interact with the estrogen receptors ERα and ERβ and can cause hormonal disorder [10]. The most sensitive species for these effects are pigs, in which ZEN administration causes alteration of the reproductive tract and decreases fertility, for example [11]. The sensitivity can be attributed to the extensive reductive metabolism to α-ZEL which has a much higher binding affinity relative to ZEN and is the predominant contributor to total estrogen receptor ligand activity after oral dosing of juvenile female pigs with ZEN [12]. Formation of α-ZEL was also observed in vitro by investigation of ZEN metabolism in Caco-2 cells; β-ZEL and several glucuronides and sulfates were also formed [13]. Additionally, in epidemiological studies a chronical exposure of ZEN was associated with precocious development of children [14,15]. Besides its estrogenic activity, ZEN is also an immunotoxic compound. In 2014 Hueza et al. showed that ZEN can modulate most aspects of immune response and impair lymphoid organs, resulting in thymus atrophy [16]. Additionally, ZEN can be considered as a clastogenic compound. The induction of chromosome aberrations was shown in vitro in HeLa cells and in vivo in mouse bone marrow cells. The proposed mechanism for the clastogenic effect is a CYP-mediated formation of catechols that can be oxidized to quinones that undergo redox cycling [17]. In human breast cancer cells (MCF-7 cells) ZEN stimulates cell proliferation and the authors conclude a possible contribution of ZEN to the increasing incidence rates of breast cancer [18]. Nevertheless, the acute toxicity of ZEN is low and evidences for carcinogenic effects are not classifiable, because of inadequate evidence in human and limited evidence in experimental animals [19].

In present ZEN regulations, the conjugated metabolites as well as its reductive forms are not comprised. In vitro analyses of the gastrointestinal digestive process showed no cleavage of ZEN conjugates, but in human microbiota fermentation the conjugates were cleaved by the microbial enzymes [5,20]. Thus, ZEN uptake might be underestimated, due to the release of absorbable ZEN. Recently, the EU-CONTAM Panel found it appropriate to set a group tolerable daily intake (TDI) for ZEN and its modified forms [21]. It must be considered that the estrogenic potency of ZEN derivatives differs. Potency factors assigned to these derivatives by EFSA CONTAM Panel are 0.2 for β-ZEL and 60 for α-ZEL relative to ZEN. Moreover, for sulfate and glucoside conjugates the same factors as for the free form are proposed. To obtain more data on the occurrence of ZEN metabolites in food and feed, standard substances are needed. Until now, some synthetic and biosynthetic strategies were developed for production of ZEN glucosides and ZEN-14-S. Chemical synthesis of ZEN-14-S and ZEN-16-G were conducted by Mikula et al. [22,23]. A procedure for selective monosulfation of ZEN on position C-14 was established by applying a 2,2,2-trichloroethyl protection. Triisopropyl-protected ZEN was used as intermediate for subsequent regiocontrolled glucosylation of ZEN on position C-16. ZEN-14-G was biosynthesized by a genetically modified yeast strain, expressing the *Arabidopsis thaliana* UDP-glucosyltransferase UGT73C6 [6]. A biosynthetic strategy for synthesis of mono- and di-glucosides of ZEN by recombinant barley glucosyltransferase HνUGT14077 was developed by Michlmayr et al. [24]. In sum, biotransformation of ZEN in plants is mainly catalyzed by UGTs and ZEN glucosides are formed. In phase II metabolism of fungi the biotransformation of ZEN to both, sulfate and glucoside conjugates, was observed. There is clear evidence that not only plant UGT can catalyze the conjugation of ZEN. Especially for fungi the ability to detoxify mycotoxins produced by co-occurring species is crucial for their survival. However, the full potential of microorganisms has not been elucidated. Thus, various species of the genera *Rhizopus* and *Aspergillus* which are known to glucosylate and or sulfatize ZEN are investigated in more detail.

**Figure 1:**
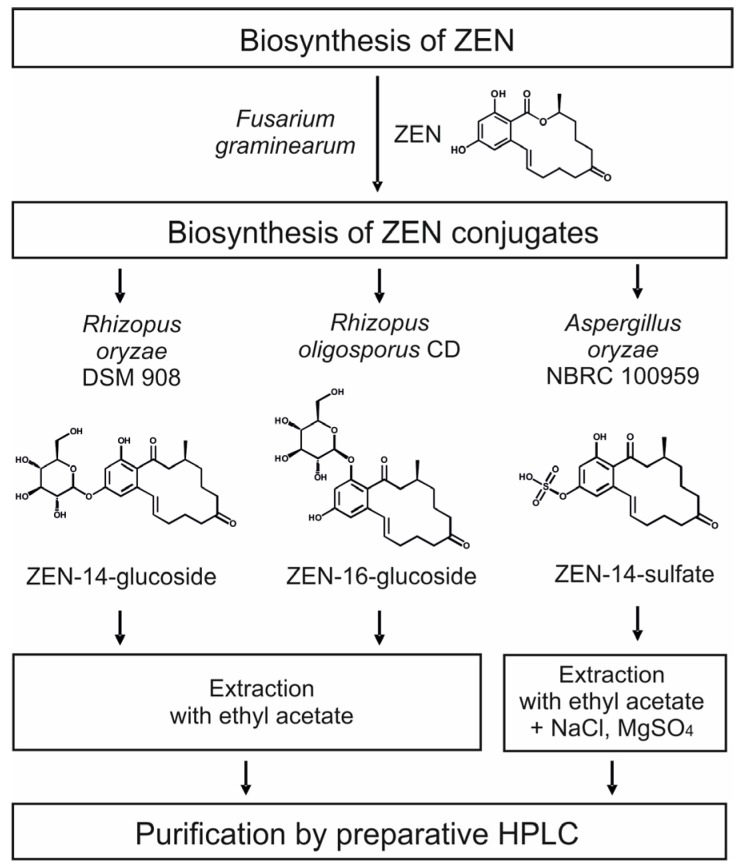
Chart of biosynthesis, isolation and purification of zearalenone (ZEN)-14-glucoside, ZEN-16-glucoside and ZEN-14-sulfate.

As ZEN conjugates are either very expensive or not commercially available until now one main goal of the present study was to develop a simple and economic method for biosynthesis of ZEN conjugates without the need of special laboratory equipment. In 2014, *Rhizopus* and *Aspergillus* strains were already identified to be capable of ZEN conjugate formation after ZEN addition [7]. Based on that data, a consecutive biosynthesis was outlined for the current study (Figure 1). In a first step, ZEN was supposed to be biosynthesized by *F. graminearum* and secondly the produced ZEN could be used for the formation of ZEN-14-S, ZEN-14-G and ZEN-16-G by selected *Rhizopus* and *Aspergillus* species under defined conditions.

## 2. Results

### 2.1. In Vitro Screening of Conjugate Producing Aspergillus and Rhizopus Species

**Figure 2:**
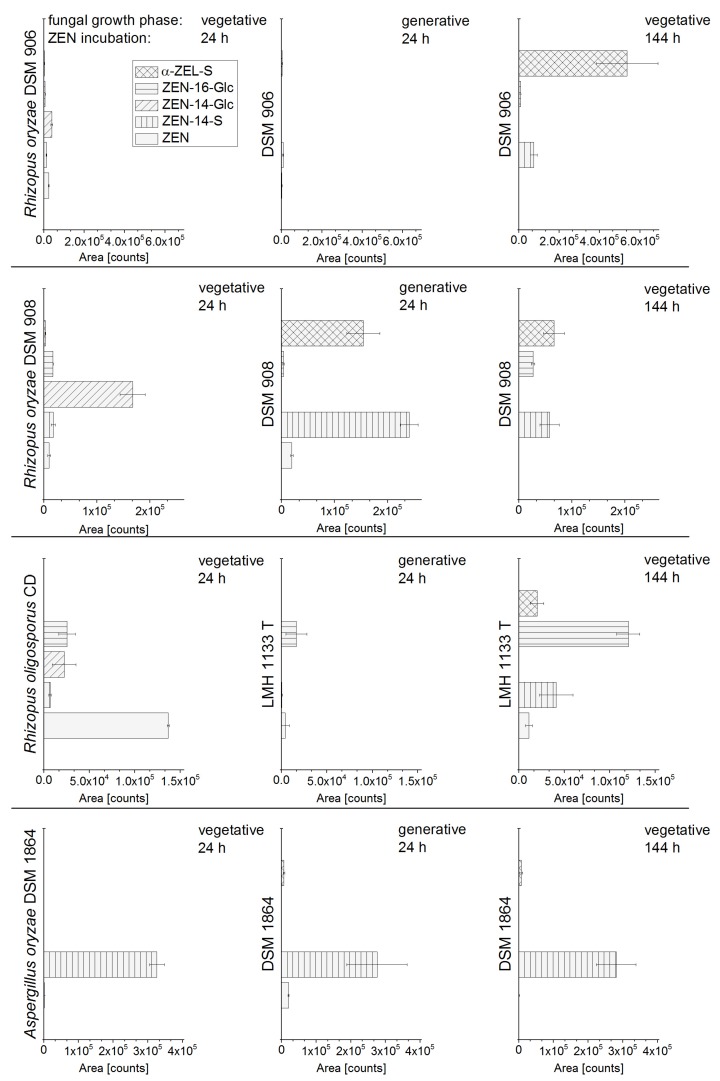
Formation of ZEN-14-sulfate (ZEN-14-S), ZEN-14-glucoside (ZEN-14-Glc), ZEN-16-glucoside (ZEN-16-Glc), and α-ZEL-sulfate (α-ZEL-S) by *R. oryzae* DSM 906, *R. oryzae* DSM 908, *R. oligosporus* CD, and *A. oryzae* DSM 1864 after ZEN addition to vegetative and generative fungal growth phase with subsequent ZEN incubation for 24 h (column 1 and 2) or 144 h (column 3); a response factor for ZEN-14-S/ZEN of 11 and for α-ZEL-S/ZEN of 2.3 was applied.

As already reported, fungal strains which convert ZEN into its glucosides and sulfates show a heterogeneous product pattern [7]. Thus, such strains that conjugate ZEN most selectively into ZEN-14-S, ZEN-14-G or ZEN-16-G were characterized in the present study. Therefore, eight fungal strains (Table 1) of the genera *Rhizopus* and *Aspergillus* were screened for ZEN conjugate formation after ZEN addition to the liquid culture; time of harvest and ZEN addition to different fungal growth phases were varied. The analyzed strains showed a very diverse metabolite pattern. In particular, the formation of the conjugates strongly varied depending on the time of harvest (ZEN incubation time) and the growth phase of the fungus, in which ZEN was added. The vegetative and generative growth stages of the various fungi were reached by 24 h and 144 h cultivation in liquid media prior to ZEN addition. Four representative fungal strains (*R. oryzae* DSM 906, *R. oryzae* DSM 908, *R. oligosporus* CD, and *A. oryzae* DSM 1864) with a diverse metabolite formation and pronounced change of the metabolite pattern dependent on different time of harvest and reproductive growth phase are shown in Figure 2. The metabolite pattern of *R. oryzae* DSM 907, *R. stolonifer* DSM 855, *R. microsporus* var. *chinensis* DSM 1834, and *A. oryzae* NBRC 100959 are shown in the Figure S1. To point out the effect of growth phase on the fungal metabolite pattern, in Figure 2, the metabolite formation after 24 h of ZEN incubation were compared for both phases. The importance of the ZEN incubation time was shown exemplarily for two time points (24 h, 144 h) after ZEN addition to the vegetative growth phase.

As described by Brodehl et al. [7] a response factor of 11 reflecting the significantly enhanced molar response in the detection for ZEN-14-S compared to ZEN was applied for better illustration of the metabolite formation obtained by HPLC-ESI-MS/MS measurements. Accordingly, for an approximate quantitative assessment of the α-ZEL-sulfate (α-ZEL-S) formation compared to ZEN, in Figure 2 a response factor of 2.3 was applied, which is derived from a factor of 16 for the α-ZEL-sulfate (α-ZEL-S) response compared to α-ZEL [7] and a response factor of 1/7 for α-ZEL compared to ZEN. The ZEL-S was identified as the isomer α-ZEL-S by enzymatic hydrolysis conducted according to Brodehl et al. [7]. Qualitative identification of ZEN and the ZEN metabolites were conducted based on a comparison of the retention time of standard substances and self-synthesized standards.

**Figure 3:**
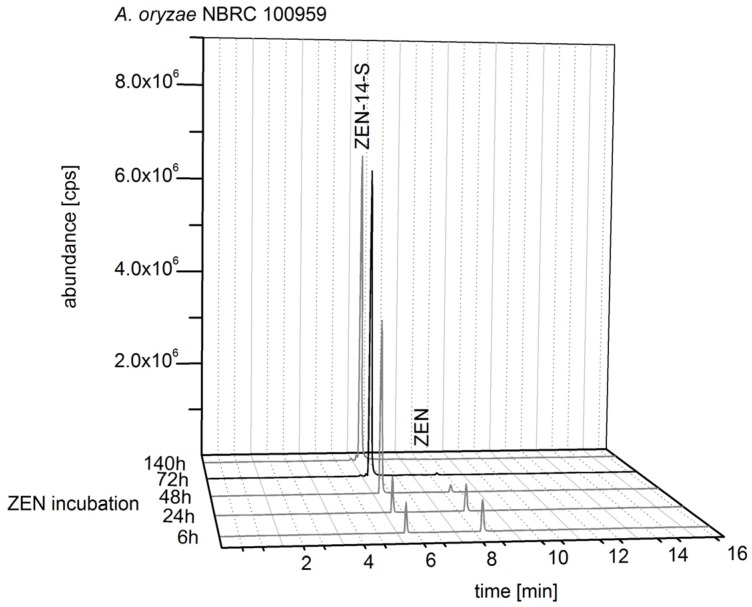
Reconstructed total ion chromatogram (TIC) with mass transitions of zearalenol (ZEL), ZEL-sulfate, ZEL-glucoside, zearalenone (ZEN), ZEN-14-sulfate (ZEN-14-S), and ZEN-glucoside analyzed in liquid media of *Aspergillus oryzae* NBRC 100959 incubated with ZEN.

The analyzed *A. oryzae* strain DSM 1864 formed primarily the ZEN-14-S, and α-ZEL-S to a small extent; for *A. oryzae* NBRC 100959 the results were the same (Figure 3). Also, for *R. microsporus* var. *chinensis* DSM 1834 only the formation of ZEN-14-S and α-ZEL-S was shown. For all aforementioned strains, no glucoside conjugates were formed (Figure S1). The ZEN metabolite formation strongly differs between the closely related strains *R. oryzae* DSM 906 and DSM 908 depending on the time of harvest. After ZEN addition to the vegetative growth phase with subsequent incubation for 24 h, DSM 908 predominantly formed the ZEN-14-G; DSM 908 media sampled after 144 h contained ZEN-16-G, α-ZEL-S and ZEN-14-S, but no ZEN-14-G. In contrast, DSM 906 showed no considerable metabolite formation after 24 h of ZEN incubation in vegetative phase, but a strong formation of the α-ZEL-S after 144 h. The results of *R. oryzae* DSM 907 and *R. stolonifer* DSM 855 resemble to those detected for DSM 908 with only minor differences.

ZEN conjugate formation was analyzed for ZEN addition to the vegetative growth phase (fungal pre-cultivation: 24 h) and for ZEN addition to generative growth phase (fungal pre-cultivation: 144 h). Especially, *R. oryzae* DSM 908 showed a completely other metabolite spectrum, when ZEN was added to the fungal culture in the vegetative phase or in the generative growth phase. For both phases, the DSM 908 liquid culture was incubated for 24 h with ZEN (Figure 2 line 2, column 1 and 2). ZEN addition to the vegetative phase resulted in a formation of ZEN-14-G and the addition of ZEN to the generative phase showed a strong formation of sulfate conjugates. Interestingly, ZEN-16-G, which is only a byproduct in the metabolite pattern of the other analyzed fungi, was the major component following 144 h of ZEN incubation in the vegetative phase of *R. oligosporus* CD. For *R. oryzae* DSM 906, *R. oligosporus* CD and *A. oryzae* DSM 1864 comparison of metabolite formation in different growth phases of the fungi is shown in Figure 2 (column 1 and 2).

In sum, ZEN addition to different fungal growth phases had a strong impact on the conjugate formation of *Rhizopus* species, but no impact for *Aspergillus oryzae* incubations. Most of the analyzed *Rhizopus* strains formed sulfate conjugates as major metabolites, when ZEN was added to generative phase and further incubated for more than six hours. Glucoside formation, especially ZEN-14-G, was predominantly observed in *Rhizopus* cultures in the first 24 h of incubation after ZEN addition to vegetative phase. However, metabolism of fungi is influenced by many external factors. Therefore, no predictions for conjugate formations of related fungal strains can be made.

The complete results of the eight fungal strains, which include all points of harvest for ZEN addition to both growth phases, are stated in the Figure S1.

### 2.2. Consecutive Biosynthesis of ZEN Conjugates

#### 2.2.1. Biosynthesis of ZEN

First, the formation of ZEN by the *F. graminearum* strains F1, F2, and F3 was analyzed on autoclaved and, therefore, enzyme deactivated rice media. The strains analyzed, showed a strong variation in ZEN production. *F. graminearum* F3 showed the highest ZEN formation after 32 days of incubation with 19.5 ± 9.3 mg/kg dry mass, whereas *F. graminearum* F1 produced ZEN after 32 days with very high amounts of 3005 ± 708 mg/kg dry mass. *F. graminearum* F2 had a maximum of ZEN formation after 20 days of incubation, but ZEN production was very inconsistent with ZEN amounts ranging from 34.6 up to 6483 mg/kg dry mass. Thus, for the first step of the consecutive biosynthesis, *F. graminearum* F1 was chosen for ZEN production, because the data were consistent and ZEN was produced in high amounts. In addition to ZEN, ZEN-14-S was also formed by *F. graminearum* F1 in a ratio of ZEN to ZEN-14-S of 1:0.02 up to 1:0.2.

#### 2.2.2. Biosynthesis of ZEN Conjugates

With a focus on conjugation products of unmodified ZEN as basic molecule, a consecutive biosynthesis was developed. Fungal strains with the best results for an exclusive formation of ZEN-14-S, ZEN-14-G, and ZEN-16-G were chosen: *A. oryzae* NBRC 100959, *R. oryzae* DSM 908, and *R. oligosporus* CD, respectively. ZEN biosynthesis and ZEN conjugate biosynthesis were combined by adding ZEN-contaminated rice flour obtained from incubation with *F. graminearum* F1 into liquid media with subsequent fungal incubation. In Figure 3, the HPLC-MS/MS analysis of the ZEN-14-S formation by *A. oryzae* NBRC 100959 for five time points of media harvest is shown. Incubation from 6 h to 140 h with ZEN (80 mg/L) showed a decrease of the ZEN content and an increase of ZEN-14-S formation. After 72 h of incubation *A. oryzae* is in the generative growth phase and an exclusive formation of ZEN-14-S with a negligible residual content of ZEN was observed. ZEN incubation extended to 140 h did not result in an important increase of ZEN-14-S.

**Figure 4:**
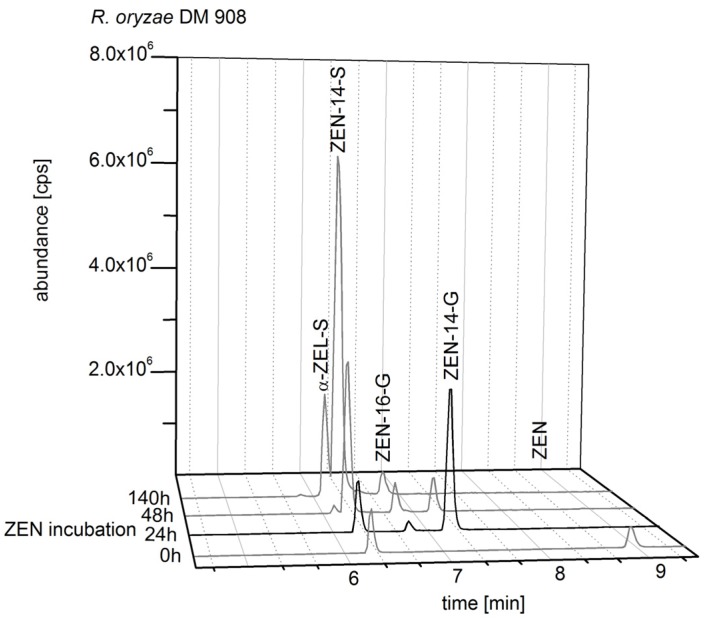
Reconstructed total ion chromatogram (TIC) with mass transitions of zearalenol (ZEL), α-ZEL-sulfate (α-ZEL-S), ZEL-glucoside, zearalenone (ZEN), ZEN-14-sulfate (ZEN-14-S), ZEN-14-glucoside (ZEN-14-G), and ZEN-16-glucoside (ZEN-16-G) analyzed in liquid media of *Rhizopus oryzae* DSM 908 incubated with ZEN.

As the ZEN conjugate formation of *R. oryzae* DSM 908 varied strongly depending on period of ZEN incubation, the most exclusive ZEN-14-G formation was achieved in the vegetative growth phase after 24 h of ZEN incubation (Figure 4). Besides to ZEN-14-G, also a marginal ZEN-16-G formation and a ZEN-14-S peak were detected after 24 h incubation with ZEN (80 mg/L). The impurity of the media with ZEN-14-S could be observed even in the beginning of the fungal incubation, because in ZEN biosynthesis by *F. graminearum* also ZEN-14-S was formed. Thus, the ZEN-14-S contamination in ZEN-14-G biosynthesis could not be avoided. However, ZEN-14-S and ZEN glucosides were separated in a subsequent cleanup step.

**Figure 5:**
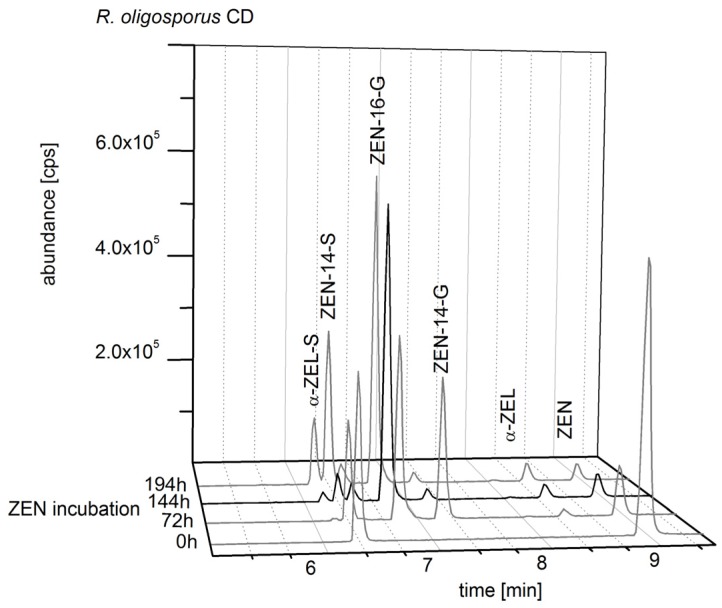
Reconstructed total ion chromatogram (TIC) with mass transitions of zearalenol (ZEL), α-ZEL-sulfate (α-ZEL-S), ZEL-glucoside, zearalenone (ZEN), ZEN-14-sulfate (ZEN-14-S), ZEN-14-glucoside (ZEN-14-G), and ZEN-16-glucoside (ZEN-16-G) analyzed in liquid media of *Rhizopus oligosporus* CD incubated with ZEN.

*R. oligosporus* CD is an appropriate producer of ZEN-16-G. In Figure 5 several time points of ZEN incubation (80 mg/L) and the resulting ZEN metabolite formation are shown. After 144 h of ZEN incubation *R. oligosporus* is in the generative growth phase and ZEN-16-G is formed as the main metabolite; the by-products which were formed only in small amounts compared to ZEN-16-G are ZEN, α-ZEL, ZEN-14-S, α-ZEL-S, and ZEN-14-G. Next to the ZEN-14-S signal, a small peak which belongs to the mass transitions of a ZEL glucoside appeared. An earlier media harvest after 72 h resulted in a smaller amount of ZEN-16-G and a higher formation of ZEN-14-G. A media harvest at a later stage, after 194 h of incubation, showed a slightly higher ZEN-16-G formation compared to 144 h of incubation, but additional a higher formation of α-ZEL-S and ZEN-14-S, and a small peak next to the ZEN-14-S signal which was allocated to the mass transitions of a ZEL glucoside.

The reconstructed total ion chromatograms in Figure 3, Figure 4 and Figure 5 showed the conjugate formation of representative samples. The analyses were conducted in triplicate. The complete results are shown in the Figures S2–S4.

### 2.3. Cleanup by Liquid–Liquid-Extraction (LLE) and Preparative Chromatography

For isolation of the ZEN conjugates the harvested media was first extracted by LLE with ethyl acetate. ZEN-14-G and ZEN-16-G were transferred into the organic phase with 99.8 ± 0.1% and 87.2 ± 0.3%, respectively. The extraction of ZEN-14-S was not sufficient with ethyl acetate (with only 50.1 ± 0.5% recovery). Therefore, the efficiency of the extraction was optimized to 88.0 ± 1.8% by adding MgSO_4_ and NaCl.

**Figure 6:**
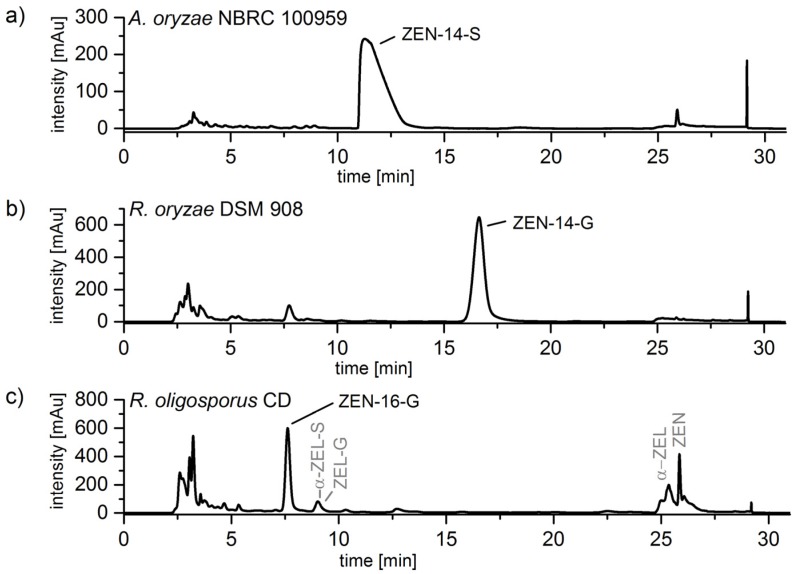
UV-chromatograms (λ = 265 nm) of the preparative separation (conducted on a Luna C18 reversed phase column; 250 mm × 21.2 mm; particle size 10 µm; pore size 100 Å) of extracted media from (**a**) *Aspergillus oryzae* NBRC 100959: Biosynthesis of zearalenone-14-sulfate (ZEN-14-S); (**b**) *Rhizopus oryzae* DSM 908: Biosynthesis of ZEN-14-glucoside (ZEN-14-G); (**c**) *Rhizopus oligosporus* CD: Biosynthesis of ZEN-16-glucoside (ZEN-16-G) with the byproducts ZEN, α-ZEL (α-zearalenol), α-ZEL-S (α-ZEL-sulfate), ZEL-G (ZEL glucoside).

In addition to the LLE, preparative chromatography was conducted as a further cleanup step. In Figure 6 the UV-chromatograms (λ = 265 nm) of the extracted media of *A. oryzae* NBRC 100959, *R. oryzae* DSM 908, and *R. oligosporus* CD are shown. The peaks of ZEN-14-S, ZEN-14-G, and ZEN-16-G with retention times of 11.3 min, 16.5 min, and 7.7 min, respectively, are well separated and after fractionating the purity of each compound was improved. Especially, the preparative purification of ZEN-16-G biosynthesis extract (Figure 6c) led to the successful separation of the byproducts ZEN (RT 25.9 min), α-ZEL (RT 25.4 min), ZEN-14-S, α-ZEL-S (9.0 min), ZEN-14-G, and the possible ZEL glucoside (9.0 min) from the target compound.

The yields of the purified analytes were 49% ZEN-14-S, 67% ZEN-14-G and 39% ZEN-16-G.

### 2.4. Structure Identification and Purity of ZEN-14-S, ZEN-14-G and ZEN-16-G

Following the described biosynthesis with subsequent cleanup ZEN-14-S, ZEN-14-G, and ZEN-16-G were obtained as a slightly yellow solid. ZEN-14-S purity of ≥97% was determined by HPLC-UV at 227 nm by calculating the percentage of the peak area in relation to total area of peaks. The HPLC-UV purity at 227 nm of ZEN-14-G was ≥96%, and for ZEN-16-G a purity of ≥69% was measured. To evaluate purity, ^1^H-NMR has also been used: purities of ≥73% ZEN-14-S, ≥82% ZEN-14-G, and ≥50% ZEN-16-G were obtained.

Structure identification was conducted by ^1^H and ^13^C-NMR. For structure elucidation, spectra with the two-dimensional methods HH-COSY, HC-HMBC, and HC-HSQC were also recorded (Figures S5–S16, Tables S1–S3). Using these 2D methods the conjugation of ZEN on position 16 with the glucose molecule could be unambiguously proven by the HMBC crosspeak of the H-17/C-16 (H at the anomeric C of glucoside to the next bound phenoxy ring-C). The assignment of C-16 could be made by an HMBC crosspeak for H-15/C-16 and due to the lack of that for H-13/C-16. The assignment of C-14 in turn can be fixed by its strong HMBC-crosspeaks to both of the adjacent aromatic hydrogen atoms H-13 and H-15. Unambiguous assignment of ZEN-14-S was conducted by comparison of the ^1^H-spectrum with the spectrum of a ZEN-14-S standard (Figure S17) chemically synthesized with the method of Mikula et al. [22].

## 3. Discussion

In this study, an easy and cost-efficient biosynthesis for ZEN-14-S, ZEN-14-G, and ZEN-16-G was developed. Fungal strains with different ZEN formation and ZEN metabolization activities were used. In the first step, we searched for a strain producing consistent and high amounts of ZEN on a low cost solid medium. *F. graminearum* F1 was pointed out as a fungal strain which produces ZEN reproducible in a mg/g range on humid rice flour. In a straightforward approach, this biosynthesis was combined with the next biosynthetical step of conjugate formation. The ZEN-contaminated flour can be directly used by adding the flour to the liquid media of conjugate biosynthesis.

For ZEN conjugate biosynthesis, fungal strains which convert ZEN to ZEN conjugates were used. In 2014, already eight fungal strains for ZEN metabolization were analyzed [7]. The results showed that the metabolite formation is very diverse even for closely related strains. In this study the in vitro screening was expanded. ZEN incubation time and ZEN addition to different growth phases of the fungi were varied. The data revealed for each ZEN conjugate one fungal strain which converts ZEN under defined conditions efficiently to ZEN-14-S, ZEN-14-G, and to ZEN-16-G (as major metabolite); yields of 49%, 67% and 39% could be achieved, respectively. Occurring losses are mainly caused by a saturation of the enzymatic reaction. The ZEN conversion rate decreases with higher initial ZEN amounts. However, there are no relevant costs for ZEN in the developed consecutive biosynthesis. Further investigations should concentrate on upscaling the production on a biotechnological scale by increase of (flask) size and volume.

The purity of ZEN-14-G, ZEN-16-G, and ZEN-14-S was determined by HPLC-UV as 96%, 69%, and 97%, respectively. Purities additionally measured by ^1^H- and ^13^C-NMR were 82% for ZEN-14-G, 50% for ZEN-16-G and 73% for ZEN-14-S. As not only UV-active substances may represent impurities originating from fungal incubation samples, for the glucosides lower purity values are detected. Caused by the cleanup, the synthesized substances contain ammonium acetate, which is very hydrophilic. Therefore, the substances also contain residual H_2_O. Additionally, the NMR spectra indicate impurities for ZEN-16-G and ZEN-14-S of small amounts (about one to two percent) of unknown substances which has a chemical structure containing a ZEN moiety.

For the very easy and fast cleanup of LLE with subsequent preparative chromatography, the results were very good. All the ZEN metabolites formed were well separated. Nevertheless, efficient strategies for chemical synthesis and biosynthesis of ZEN conjugates have already been developed. A chemical synthesis of ZEN-14-S and ZEN-16-G was conducted by Mikula et al. [22,23] and Michlmayr et al. [24] synthesized ZEN-14-G and ZEN-16-G in good purity. However, with the ^1^H-NMR purity of the conjugates known, also the standards obtained by our fungal biosynthesis can be used for accurate quantification or toxicological experiments.

One main advantage of this approach is the regioselectivity. Especially the 16-position of ZEN is difficult to conjugate in chemical synthesis, because the carbon atom is very inactive. Thus, biosynthesis is an appropriate alternative way for a targeted regioselective production of conjugated mycotoxins. While for plants it has been clarified which glucosyltransferases are responsible for the glucosylation of ZEN, the UGTs in *Rhizopus* or *Aspergillus* species are still unknown. As some *A. oryzae* species genomes have been sequenced, screening experiments could help to obtain information on which enzymes catalyze distinct conjugation reactions.

Additionally, the biosynthesis developed could be also used for biosynthesis of other metabolites like ZEL conjugates. The present results of the in vitro screening indicated the formation of a ZEL-glucoside by detection of the mass transitions and α-ZEL-S was also formed as major metabolite by *Rhizopus oryzae* DSM 906. Nevertheless, more fungal strains should be screened for an exclusive formation and the cleanup must be optimized.

## 4. Conclusions

This study shows a new approach for the synthesis of ZEN conjugates. Fungal strains were successfully utilized for an economic targeted and regioselective biosynthesis of ZEN-14-S, ZEN-14-G and ZEN-16-G. No special laboratory equipment is needed. It is easy to handle and not cost intensive. Additionally, costs for ZEN can be avoided, because ZEN biosynthesis by *F. graminearum* was combined with ZEN conjugate production by *Aspergillus* and *Rhizopus* species. Nevertheless, for the cleanup method the purities of the obtained standards are good, but especially for ZEN-16-G a further cleanup step should be conducted for improvement of its purity. 

## 5. Materials and Methods

### 5.1. Chemicals and Media

Potato dextrose agar (PDA) and potato dextrose broth (PDB) were purchased from Carl Roth GmbH + Co. KG (Karlsruhe, Germany). Rice flour was purchased from Biokorn GmbH + Co. KG (Aalen, Germany). ZEN was acquired from Tocris Bioscience (Bristol, England). A stock (1 mg mL^−1^) and working (5 µg mL^−1^) solution of ZEN was prepared as methanolic solution and stored at −20 °C. α-ZEL was purchased from Sigma-Aldrich Chemie GmbH (Steinheim, Germany). ZEN-14-S, ZEN-14-G und ZEN-16-G as reference standard were kindly provided by Prof. Franz Berthiller (University of Natural Resources and Life Sciences, Vienna, Austria). Acetonitrile and methanol were of HPLC-grade and obtained from Th. Geyer (Renningen, Germany). Ethyl acetate p. a. and sodium chloride p. a. were also purchased from Th. Geyer (Renningen, Germany). Magnesium sulfate was acquired from Sigma Aldrich (Steinheim, Germany). Ammonium acetate was purchased from Mallinckrodt Baker Inc. (Griesheim, Germany). Ultrapure water was obtained from a Seralpur PRO 90 CN purification system by Seral (Ransbach-Baumbach, Germany). Deuterated dimethyl sulfoxide (99.8 atom-% D) was acquired from Merck Switzerland. Trimesic acid trimethyl ester were purchased from OrganoSpezialChemie GmbH Bitterfeld. It’s purity has been traced back to that of NIST standard MRM 350b by the inhouse ^1^H qNMR method.

### 5.2. HPLC-MS/MS Analysis

HPLC-MS/MS (high-performance liquid chromatography hyphenated to tandem mass spectrometry) analysis was performed on a 1100 series HPLC system from Agilent Technologies (Waldbronn, Germany) connected to an API 4000 triple-quadrupole MS/MS system from Sciex (Framingham, MA, USA). The analytical column was a Synergi Polar-RP (150 mm × 3.0 mm, paricle size 4 µm, pore size 80 Å) in combination with a corresponding guard column (Phenomenex, Aschaffenburg, Germany). The column temperature was set to 30 °C. Solvent A was water with 5 mM ammonium acetate and solvent B acetonitrile/water (99:1; *v*/*v*) with 5 mM ammonium acetate. The gradient used was as follows: 0–2 min isocratic with 10% B, 2–4 min linear to 40% B, 4–10 min linear to 100% B, isocratic 10–13 min 100% B, shifting back to 10% B and reconditioning from 13–17 min. The flow rate of the mobile phase was 0.7 mL/min and 10 µL was used as standard injection volume. The ESI interface was operated in negative ionization mode at 450 °C with the following settings: curtain gas 20 psi, nebulizer gas 60 psi, heater gas 60 psi, ionization voltage −4500 V. MS/MS measurements were exclusively conducted in selected reaction monitoring (SRM) mode. Two mass transitions were recorded for each analyte: ZEN *m/z* 317.0 → 130.8 (declustering potential (DP) −15 V, collision energy (CE) −40 eV), *m/z* 317.0 → 174.8 (DP = −15 V, CE = −30 eV); ZEN-sulfate *m/z* 397.1 → 317.1 (DP = −65 V, CE = −30 eV), *m/z* 397.1 → 175.0 (DP = −65 V, CE = −50 eV); ZEN-glucoside *m/z* 479.1 → 317.0 (DP = −65 V, CE = −16 eV), *m/z* 479.1 → 130.8 (DP = −65 V, CE = −50 eV); ZEL *m/z* 319.2 → 174.0 (DP = −30 V, CE = −30 eV), *m/z* 319.2 → 160.0 (DP = −75 V, CE = −30 eV); ZEL-sulfate *m/z* 399.2 → 319.2 (DP = −30 V, CE = −30 eV), *m/z* 399.2 → 275.2 (DP = −30 V, CE = −40 eV); ZEL-glucoside *m/z* 481.2 → 319.2 (DP = −65 V, CE = −16 eV), *m/z* 481.2 → 275.2 (DP = −65 V, CE = −30 eV).

### 5.3. In Vitro-Screening of Aspergillus and Rhizopus Species

Eight different fungal strains of the genera *Rhizopus* and *Aspergillus* (see Table 1) were screened for ZEN conjugate formation; ZEN addition to different fungal growth phases and time of harvest were varied.

In the beginning of fungal growth, only mycelia and no spores are produced (vegetative growth phase). Dependent on the fungal strains mycelia production stops after 2–4 days and the fungus starts sporulation (generative growth phase). Change of growth phase was detected by visual investigation of sporulation and detection of the pH value which changes from pH 3 (vegetative growth phase) to pH 7 (generative growth phase). ZEN conjugate formation was analyzed for ZEN addition to the vegetative growth phase (fungal incubation: 24 h) and for ZEN addition to generative growth phase (fungal incubation: 144 h).

Cultivation for biotransformation were conducted as follows: Liquid media (50 mL PDB) in 250-mL Erlenmeyer flasks were inoculated with pieces of mycelia. Fungal incubation was conducted for 24 h or 144 h at 30 °C in a New Brunswick Scientific Innova^®^ 44 rotary shaker set to 150 rpm (Eppendorf AG, Hamburg, Germany). To each liquid culture 1 mL working solution of ZEN was added and incubation was continued for 6, 12, 24, 48, 72, 144 and 192 h. After incubation period, an aliquot of 1 mL was transferred to a 1.5-mL Eppendorf tube and centrifuged at 16,200× *g* for 10 min with an Eppendorf centrifuge 5415 R (Eppendorf AG, Hamburg, Germany). For protein precipitation, 500 µL ice-cold acetonitrile was added to 500 µL supernatant, stored over night at 4 °C and centrifuged at 11,500× *g* for 5 min with an Eppendorf MiniSpin Plus (Eppendorf AG, Hamburg, Germany). The supernatant was analyzed by HPLC-MS/MS.

### 5.4. Biosynthesis of ZEN

Three strains of *F. graminearum* were screened for ZEN production (see Table 2). Stock cultures were grown on potato dextrose agar (PDA) for 14 days at 23 °C. According to Plasencia and Mirocha which have shown a ZEN production by *F. graminearum* of 1.2 g/kg on rice, rice was chosen as incubation media in this study as well [2]. 30 g rice flour and 15 mL distilled water were added to 250-mL Erlenmeyer flask. The flask was closed with a cellulose plug and autoclaved for 20 min at 121 °C. The autoclaved media was inoculated with pieces of mycelia and incubated at 23 °C for 5, 10, 15, 20, 25 and 32 days. For 20, 25 and 32 days analyses in triplicate were conducted. ZEN biosynthesis was stopped by autoclaving the fungal incubation. Subsequently, the content of the flask was freeze dried and powdered. Analyzation of the ZEN amount was conducted by extraction of 2 g flour with 20 mL acetonitrile/water (80:20 *v*/*v*) for 3 h with 1/300 min at a horizontal shaker HS 501 digital (IKA^®^, Staufen, Germany). The supernatant was used for direct analysis by HPLC-MS/MS (see Section 5.2).

As described by Brodehl et al. [7] ZEN-14-S was determined using relative response factor of 11 for ZEN-14-S to ZEN. Response factor was estimated by comparing the MS/MS peak area before and after quantitative sulfate ester cleavage. ZEN was determined by external calibration using the commercially available standard substances

### 5.5. Biosynthesis of ZEN Conjugates

ZEN biosynthesis was coupled with ZEN conjugate biosynthesis by adding ZEN-contaminated rice flour into liquid media with subsequent fungal incubation. *A. oryzae* NBRC 100959, *R. oryzae* DSM 908 and *R. oligosporus* CD were utilized for biosynthesis of ZEN-14-S (incubation parameter: 72 h, 150 rpm, 30 °C), ZEN-14-G (incubation parameter: 24 h, 150 rpm, 30 °C) and ZEN-16-G (incubation parameter: 144 h, 150 rpm, 30 °C), respectively. ZEN-contaminated rice flour produced by *F. graminearum* F1 (see Section 5.3.) with a total amount of 4 mg ZEN was added to 50 mL PDB in a 250-mL Erlenmeyer flask (c(ZEN) = 80 mg/L).

Prior to this, ZEN conversion in consecutive biosynthesis was analyzed by screening over a period of 9 days. The ZEN containing liquid media was autoclaved (120 °C, 20 min), inoculated with pieces of mycelia and incubated at 30 °C in a New Brunswick Scientific Innova^®^ 44 rotary shaker set to 150 rpm Sampling was conducted after 0, 6, 24, 48 72, 140, 192 and 216 h. Analyzation of the media samples was conducted analogues to media samples of the in vitro screening.

### 5.6. Liquid–Liquid-Extraction (LLE) and Preparartive Chromatography

The liquid fungal culture of ZEN conjugate biosynthesis was centrifuged at 3101 *g* for 10 min with a Sigma 6K15 centrifuge (Sigma-Aldrich GmbH, Steinheim, Germany). The supernatant was extracted by LLE. 20 mL of liquid media were extracted three times was 20 mL ethyl acetate on a horizontal shaker with 1/300 min for 30 min. The extract was evaporated to dryness under a steam of nitrogen and subsequently dissolved in H_2_O/ACN (70/30; *v*/*v*) and filtered with Chromavil^®^ A-20/25 syringe filter (pore size 0.2 µm, diameter 25 mm) purchased from Macherey-Nagel (Düren, Germany). For ZEN-14-S the procedure of LLE was modified for improvement of extraction efficiency. Before ethyl acetate extraction 2 g of sodium chloride and 8 g of magnesium sulfate were added to the media and cooled in ice water for 2 min. Subsequently, media extraction is conducted as described above.

The concentrated extract was further purified by preparative chromatography. The HPLC was equipped with a fraction collector, a diode array detector (DAD) and a single quadrupole MS (Agilent Technologies 6130 Quadrupole LC/MS). HPLC-DAD analyses were conducted using an Agilent 1200 series HPLC (Agilent Technologies, Waldbronn, Germany). HPLC separation of ZEN and their metabolites was conducted on a Luna C18 (250 mm × 21.2 mm, particle size 10 µm, pore size 100 Å) reversed phase column (Phenomenex, Aschaffenburg, Germany) in combination with a corresponding guard column. The column temperature was set to 30 °C and the injection volume was 500 µL. The following gradient was applied with a flow rate of 20 mL/min: 0–22 min isocratic with 30% B, then from 22–22.5 min switched linear to 100% B, following by a wash out step of 2 min from 22.5–26 min and after shifting back to 30% B from 26–26.5 min a reconditioning step from 26.5–31 min followed. Mobile phase A was water with 5 mM ammonium acetate and mobile phase B acetonitrile/water (99:1; *v*/*v*) with 5 mM ammonium acetate. The ESI interface was operated in negative-ionization mode at 250 °C with the following settings: nebulizer gas 35 psi, ionization voltage −3000 V. MS measurements were conducted in scan mode 100–700 *m/z*. The compounds were collected by mass-based fractionation set to *m/z* 397.1 for ZEN-14-S and *m/z* 479.1 for ZEN-14-G and ZEN-16-G.

### 5.7. NMR Analysis

Confirmation of ZEN-14-S, ZEN-14-G, and ZEN-16-G structure was conducted by nuclear magnetic resonance spectroscopy (NMR). NMR spectra were recorded in DMSO-d_6_ on a Bruker Avance 600 MHz WB NMR spectrometer (Bruker BioSpin GmbH, Rheinstetten, Germany) equipped with a 5 mm BBI 600 MHz W2 (Z-gradient, BTO) probehead for ^1^H and 2D measurements as well as a 5 mm BBO 600 MHz W2 (BTO) probehead for ^13^C NMR, operating at 600.2 MHz for ^1^H and 150.9 MHz for ^13^C. Data were recorded using TopSpin 2.1 and evaluated by means of TopSpin 3.1 software (Bruker BioSpin GmbH, Rheinstetten, Germany). Chemical shifts were established based on ^1^H and ^13^C signals of TMS = 0 ppm (tetramethylsilane). To evaluate the purities of the synthesized standards ^1^H-qNMR were conducted using trimesic acid trimethyl ester as standard.

### 5.8. Purity of ZEN-14-S, ZEN-14-G and ZEN-16-G

The purity was determined by HPLC-UV. The analyses were performed on a system from Agilent Technologies (Waldbronn, Germany) with a 1260 infinity quaternary pump, a 1100 series autosampler and a 1200 series column oven connected to a 1200 series DAD. The analytical column was a Gemini C18-NX (150 mm × 2.0 mm, paricle size 3 µm, pore size 110 Å) in combination with a corresponding guard column (Phenomenex, Aschaffenburg, Germany). The column temperature was set to 35 °C. Solvent A was water with 5 mM ammonium acetate and solvent B acetonitrile/water (99:1; *v*/*v*) with 5 mM ammonium acetate. The gradient used was as follows: 0–3 min isocratic with 10% B, switch from 3–3.1 min to 25% B, 3.1–16 min isocratic with 25% B, 16–25 min linear to 100% B, isocratic 25–29 min 100% B, shifting back to 10% B from 29–29.1 min and reconditioning from 29.1–36 min. The flow rate of the mobile phase was 0.25 mL/min and 20 µL was used as standard injection volume.

## Figures and Tables

**Table 1:** Fungal strains of in vitro screening.

Species	Designation	Source
*Rhizopus oryzae*	DSM 906	DSMZ *
*Rhizopus oryzae*	DSM 907	DSMZ *
*Rhizopus oryzae*	DSM 908	DSMZ *
*Rhizopus stolonifer*	DSM 855	DSMZ *
*Rhizopus microsporus* var. *chinensis*	DSM 1834	DSMZ *
*Rhizopus oligosporus*	CD (LMH 1133 T)	Hering et al. [25]
*Aspergillus oryzae*	DSM 1864	DSMZ *
*Aspergillus oryzae*	NBRC 100959	Nite Biological Resource Center (Tokio, Japan)

* German Collection of Microorganisms and Cell Cultures (Braunschweig, Germany).

**Table 2:** *Fusarium graminearum* strains used for ZEN production screening [26].

Fungal Strain	Isolate	Isolated by
*F. graminearum* F1	37	Gossmann, HU Berlin 1994
*F. graminearum* F2	09-53b	Pogoda, Luxemburg 2009
*F. graminearum* F3	MUCL 11946	Kinnard, Belgien 1969

## Supplementary Materials

The following are available online at http://www.mdpi.com/2072-6651/10/3/104/s1, Table S1: ^1^H and ^13^C NMR shifts of ZEN-14-G, Table S2: ^1^H and ^13^C NMR shifts of ZEN-14-S, Table S3: ^1^H and ^13^C NMR shifts of ZEN-16-G, Figure S1: Formation of ZEN-14-sulfate, ZEN-14-glucoside, ZEN-16-glucoside and α-ZEL-sulfate by *Rhizopus oryzae* DSM 906, DSM 907 and DSM 908, *R. oligosporus* CD, *Aspergillus oryzae* DSM 1864, *A. oryzae* NBRC 100959, *R. microsporus* var. *chinensis* DSM 1834 and *R. stolonifer* DSM 855 after addition of 1 mL ZEN solution (c = 5 µg/mL) to 50 mL fungal culture in vegetative and generative growth phase and subsequent ZEN incubation over a period of 10 to 12 days, Figure S2: Formation of ZEN-14-sulfate, ZEN-14-glucoside, ZEN-16-glucoside and α-ZEL-sulfate by *Aspergillus oryzae* NBRC 100959 after addition of ZEN-contaminated rice flour (containing four milligrams of ZEN) to 50 mL potato dextrose liquid media and subsequent fungal incubation over a period of nine days, Figure S3: Formation of ZEN-14-sulfate, ZEN-14-glucoside, ZEN-16-glucoside and α-ZEL-sulfate by *Rhizopus oryzae* DSM 908 after addition of ZEN-contaminated rice flour (containing four milligrams of ZEN) to 50 mL potato dextrose liquid media and subsequent fungal incubation over a period of nine days, Figure S4: Formation of ZEN-14-sulfate, ZEN-14-glucoside, ZEN-16-glucoside and α-ZEL-sulfate by *Rhizopus oligosporus* CD after addition of ZEN-contaminated rice flour (containing four milligrams of ZEN) to 50 mL potato dextrose liquid media and subsequent fungal incubation over a period of nine days, Figure S5: ^1^H-qNMR spectrum of ZEN-14-G in DMSO-d6; standard: trimesic acid trimethyl ester, Figure S6: HH-COSY spectrum of ZEN-14-G, Figure S7: HC-HSQC spectrum of ZEN-14-G, Figure S8: HC-HMBC spectrum of ZEN-14-G, Figure S9: ^1^H-qNMR spectrum of ZEN-14-S in DMSO-d6; standard: trimesic acid trimethyl ester, Figure S10: HH-COSY spectrum of ZEN-14-S, Figure S11: HC-HSQC spectrum of ZEN-14-S, Figure S12: HC-HMBC spectrum of ZEN-14-S, Figure S13: ^1^H-qNMR spectrum of ZEN-16-G in DMSO-d6; standard: trimesic acid trimethyl ester, Figure S14: HH-COSY spectrum of ZEN-16-G, Figure S15: HC-HSQC spectrum of ZEN-16-G, Figure S16: HC-HMBC spectrum of ZEN-16-G, Figure S17: Comparison of the ^1^H-NMR spectra of the biosynthesized ZEN-14-S (above) and the chemically synthesized ZEN-14-S standard (below).
