# Supplementary material for: Biosynthesis and Characterization of Zearalenone-14-Sulfate, Zearalenone-14-Glucoside and Zearalenone-16-Glucoside Using Common Fungal Strains
Source: Toxins (Basel). 2018 Mar 1;10(3):104. doi: 10.3390/toxins10030104 (PMC5869392; doi:10.3390/toxins10030104)
Supplement: Supplementary file 1 [file toxins-10-00104-s001.docx]

Supplementary Materials: Biosynthesis and Characterization of Zearalenone-14-Sulfate, Zearalenone-14-Glucoside and Zearalenone-16-Glucoside Using Common Fungal Strains

Antje Borzekowski, Tatjana Drewitz, Julia Keller, Dietmar Pfeifer, Hans-Jörg Kunte, Matthias Koch, Sascha Rohn and Ronald Maul

**Table S1.** ^1^H and ^13^C NMR shifts of ZEN-14-G.

| **position** | **^1^H (ppm)** | **mult** | **^13^C (ppm)** |
| --- | --- | --- | --- |
| 1 |  |  | 168.3 |
| 3 | 5.07 | dqd, 8.7, 6.3, 2.3 Hz | 71.5 |
| 3-CH_3_ | 1.27 | d, 6.3 Hz | 19.8 |
| 4 | 1.51, 1.65 | m | 34.2 |
| 5 | 1.60 | m | 20.9 |
| 6 | 2.28, 2.31 | m | 42.9 |
| 7 |  |  | 210.5 |
| 8 | 2.37 | ddd, 17.7, 8.0, 3.4 Hz | 36.5 |
|  | 2.46 | ddd, 17.7, 8.9, 3.4 Hz |  |
| 9 | 1.67, 1.74 | m | 20.9 |
| 10 | 2.03, 2.18 | m | 30.9 |
| 11 | 6.01 | dt, 15.5, 7.3 Hz | 132.8 |
| 12 | 6.45 | dt, 15.7, 1.5 Hz | 133.4 |
| 13 | 6.68 | d, 2.3 Hz | 105.0 |
| 14 |  |  | 159.7 |
| 15 | 6.47 | d, 2.3 Hz | 102.6 |
| 16 |  |  | 158.5 |
| 17 |  |  | 111.5 |
| 18 |  |  | 138.3 |
| 1` | 4.89 | d, 7.7 Hz | 100.1 |
| 2` | 3.22 | t, 8.1 Hz | 73.1 |
| 3` | 3.28 | t, 8.9 Hz | 76.5 |
| 4` | 3.16 | t, 9.3 Hz | 69.7 |
| 5` | 3.34 | m | 77.1 |
| 6` | 3.46 | dd, 11.6, 5.8 Hz | 60.6 |
|  | 3.69 | dd, 11.6, 1.5 Hz |  |

**Table S2.** ^1^H and ^13^C NMR shifts of ZEN-14-S.

| **position** | **^1^H (ppm)** | **mult** | **^13^C (ppm)** |
| --- | --- | --- | --- |
| 1 |  |  | 167.9 |
| 3 | 5.01 | dqd, 8.7, 6.3, 2.4 Hz | 71.1 |
| 3-CH_3_ | 1.26 | d, 6.3 Hz | 19.7 |
| 4 | 1.50, 1.64 | m | 34.2 |
| 5 | 1.54, 1.69 | m | 20.8 |
| 6 | 2.26, 2.33 | m | 43.1 |
| 7 |  |  | 210.6 |
| 8 | 2.30, 2.52 | m | 35.6 |
| 9 | 1.61, 1.80 | m | 20.9 |
| 10 | 1.99, 2.22 | m | 30.7 |
| 11 | 5.93 | ddd, 15.6, 8.6, 5.7 Hz | 132.3 |
| 12 | 6.35 | dd, 15.6, 3.1 Hz | 129.0 |
| 13 | 6.77 | d, 2.1 Hz | 108.0 |
| 14 |  |  | 155.6 |
| 15 | 6.73 | d, 2.1 Hz | 106.2 |
| 16 |  |  | 156.7 |
| 17 |  |  | 113.7 |
| 18 |  |  | 136.6 |

**Table S3.** ^1^H and ^13^C NMR shifts of ZEN-16-G.

| **position** | **^1^H (ppm)** | **mult** | **^13^C (ppm)** |
| --- | --- | --- | --- |
| 1 |  |  | 166.8 |
| 3 | 5.13 | dqd, 8.4, 6.3, 2.3 Hz | 70.7 |
| 3-CH_3_ | 1.26 | d, 6.4 Hz | 19.4 |
| 4 | 1.47, 1.65 | m | 34.1 |
|  | 1.47, 1.67 | m | 20.5 |
| 6 | 2.18 | m | 43.1 |
|  | 2.42 | ddd, 13.2, 8.7, 4.7 Hz |  |
| 7 |  |  | 210.5 |
| 8 | 2.18 | m | 36.9 |
|  | 2.56 | ddd, 17.3, 11.2, 3.7 Hz |  |
| 9 | 1.50, 1.89 | m | 20.8 |
| 10 | 1.90, 2.26 | m | 30.7 |
| 11 | 5.99 | ddd, 15.6, 9.7, 4.2 Hz | 132.5 |
| 12 | 6.15 | dd, 15.6, 1.5 Hz | 128.0 |
| 13 | 6.58 | d, 2.0 Hz | 104.5 |
| 14 |  |  | 159.1 |
| 15 | 6.51 | d, 2.0 Hz | 101.2 |
| 16 |  |  | 155.0 |
| 17 |  |  | 115.2 |
| 18 |  |  | 135.5 |
| 1` | 4.80 | d, 7.7 Hz | 100.1 |
| 2` | 3.14 | t, 8.9 Hz | 73.3 |
| 3` | 3.26 | t, 8.9 Hz | 76.9 |
| 4` | 3.16 | dd, 10.6, 9.0 Hz | 69.5 |
| 5` | 3.27 | ddd, 9.7, 5.4, 2.1 Hz | 77.0 |
| 6` | 3.49 | dd, 11.9, 5.5 Hz | 60.6 |
|  | 3.69 | dd, 11.9, 2.1 Hz |  |

| **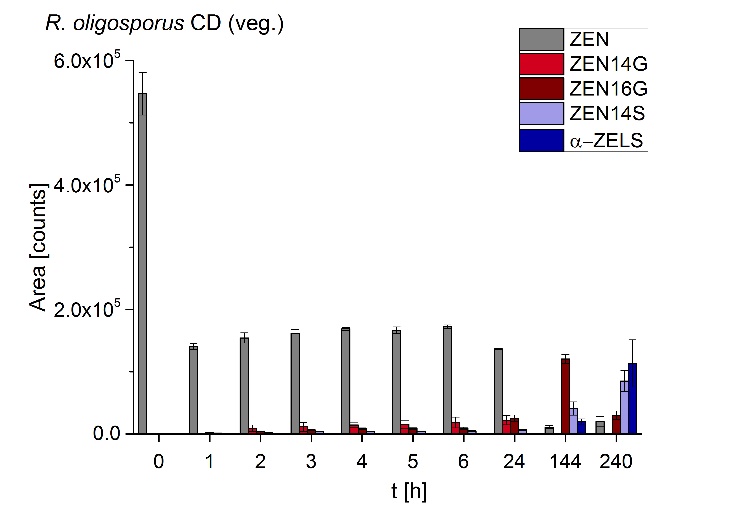** | **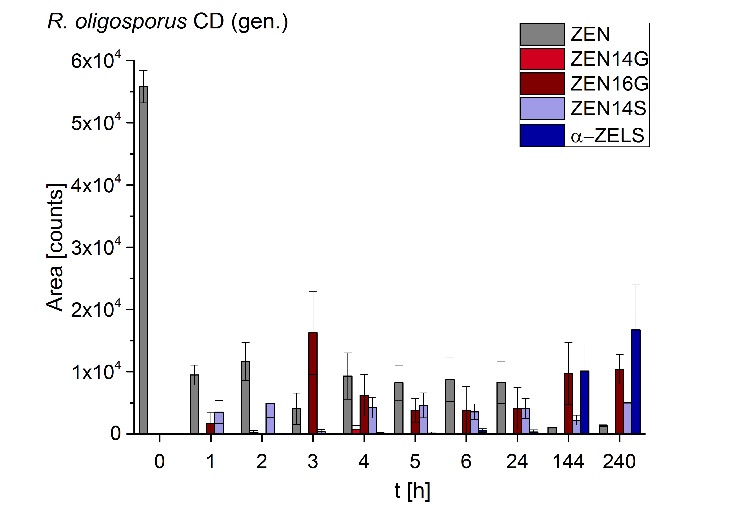** |
| --- | --- |
| **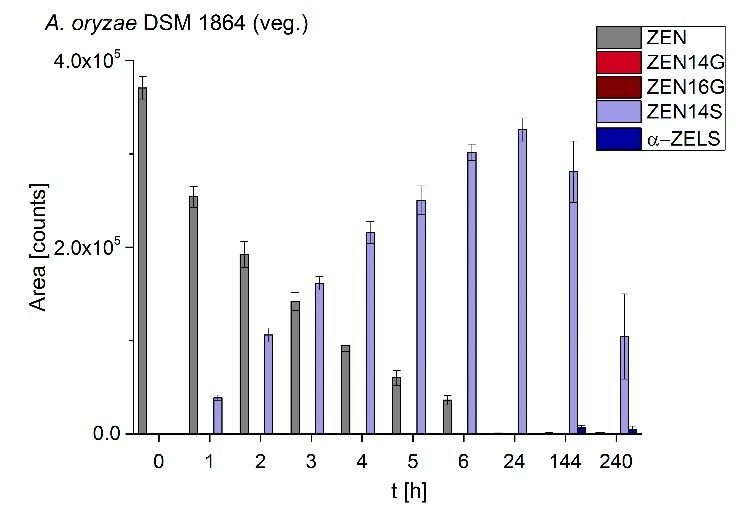** | **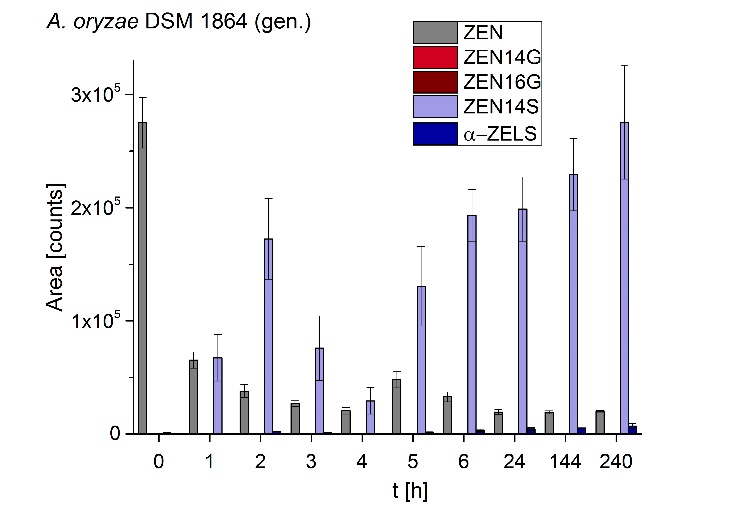** |
| **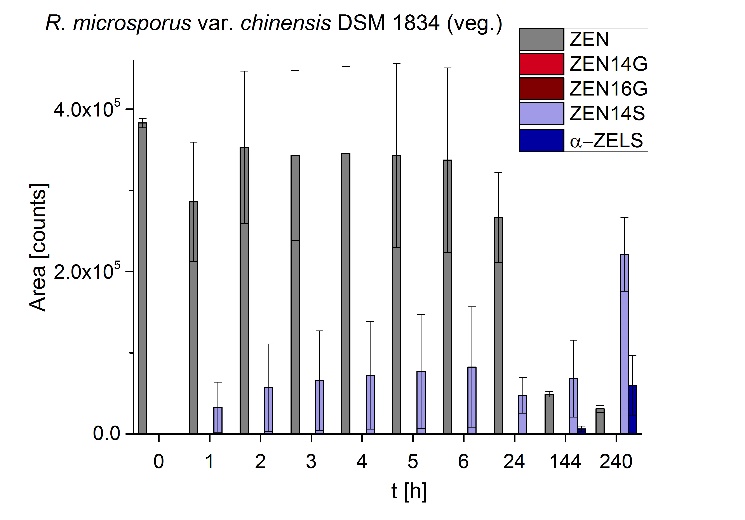** | **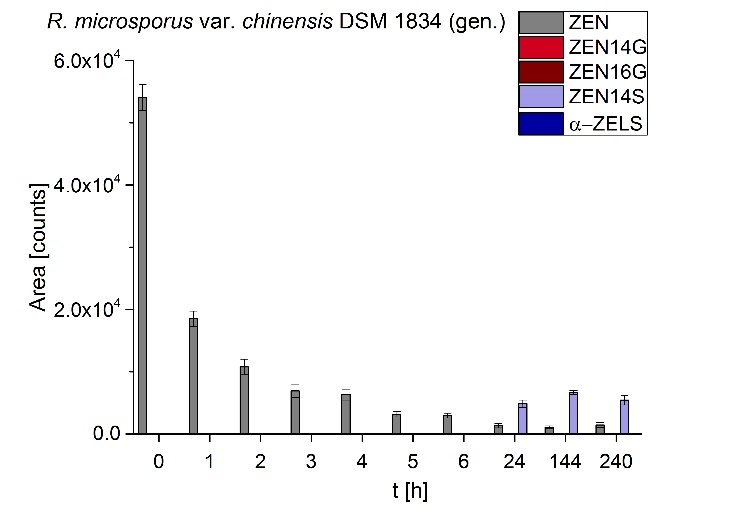** |
| **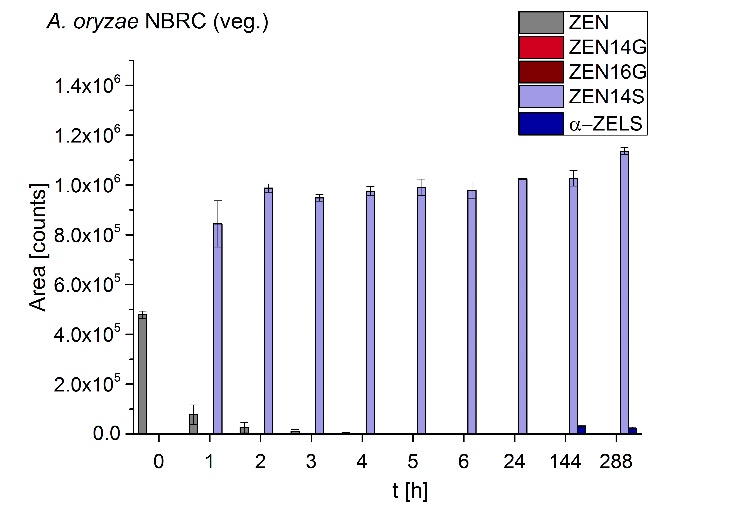** | **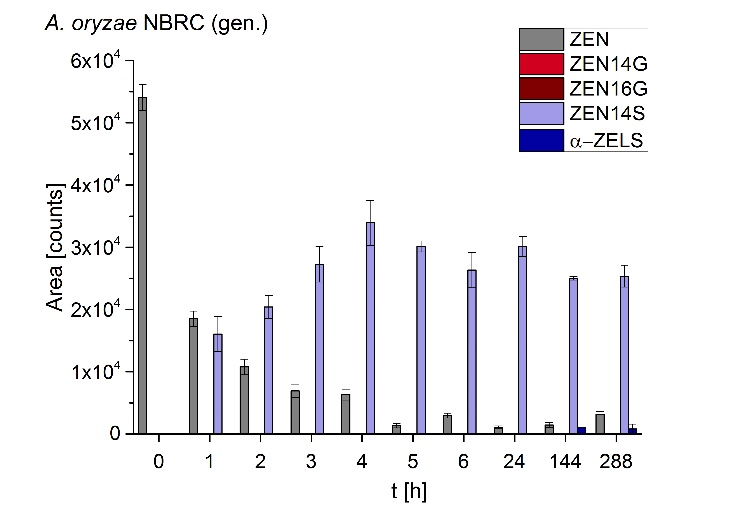** |
| **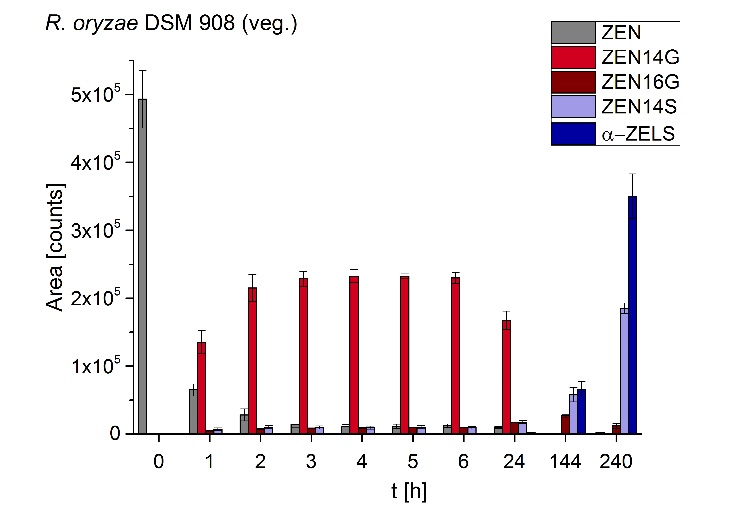** | **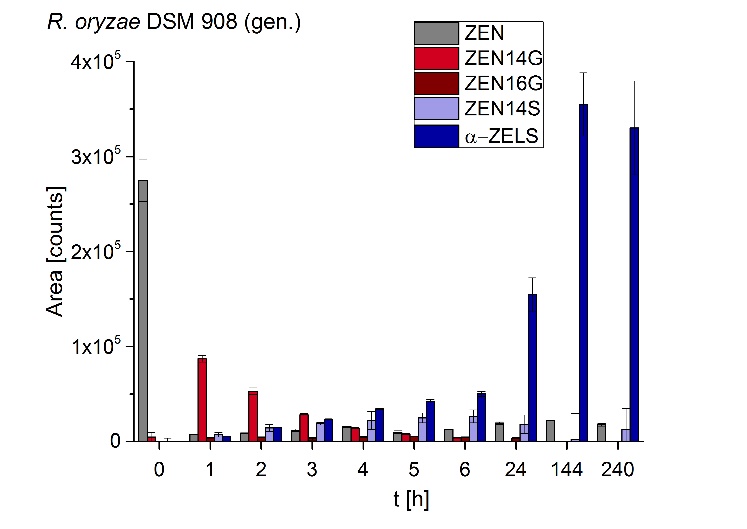** |
| **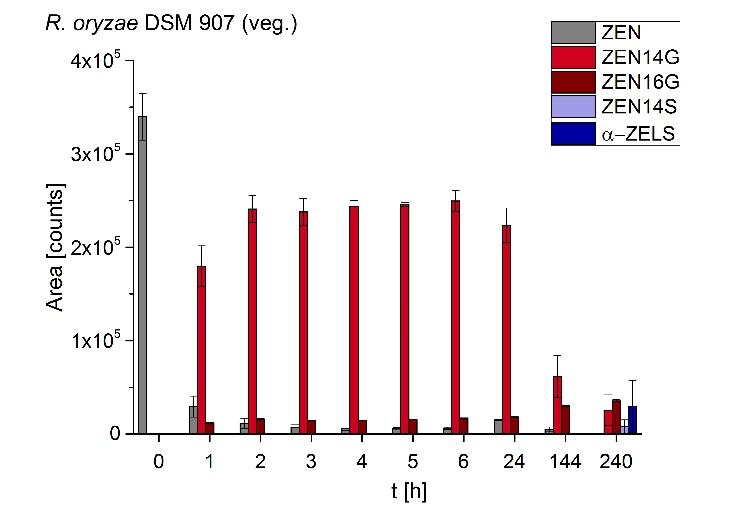** | **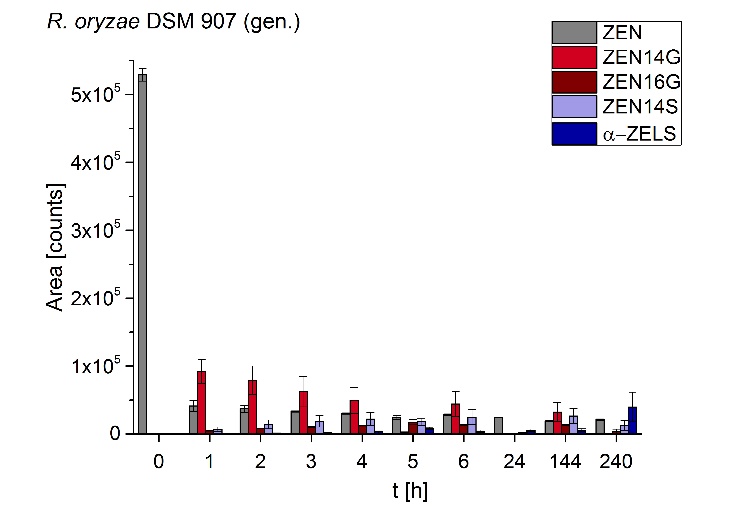** |
| **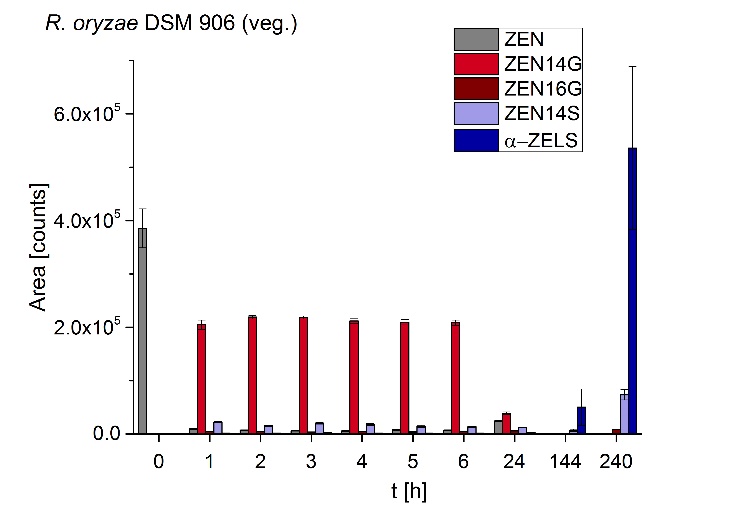** | **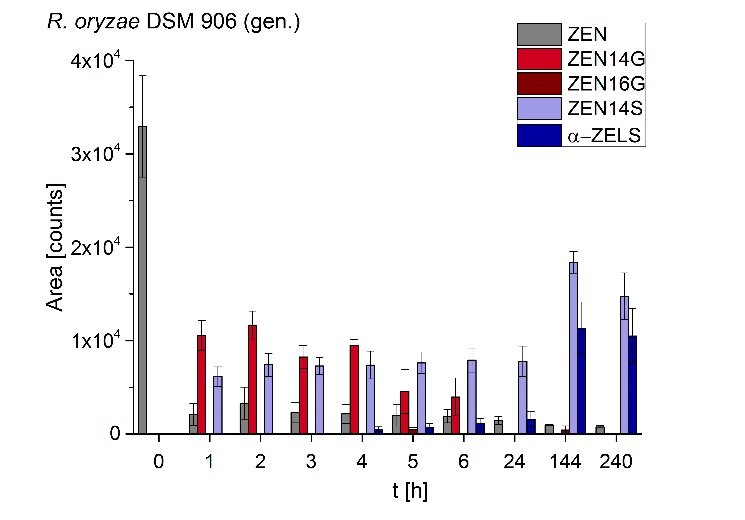** |
| **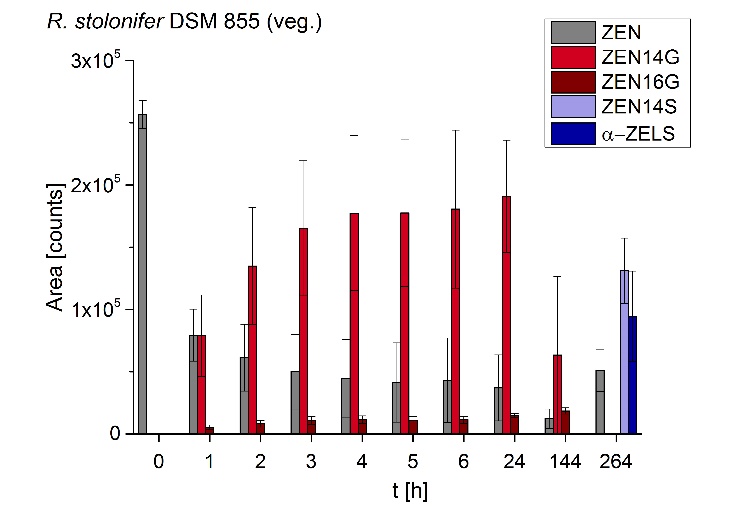** | **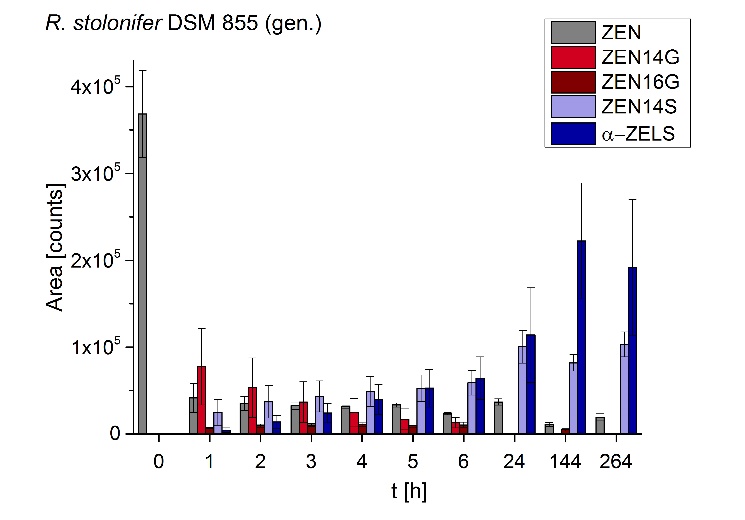** |

**Figure S1.** Formation of ZEN-14-sulfate (ZEN14S), ZEN-14-glucoside (ZEN14G), ZEN-16-glucoside (ZEN16G) and α-ZEL-sulfate (α-ZELS) by *Rhizopus oryzae* DSM 906, DSM 907 and DSM 908, *R. oligosporus* CD, *Aspergillus oryzae* DSM 1864, *A. oryzae* NBRC 100959, *R. microsporus* var. *chinensis* DSM 1834 and *R. stolonifer* DSM 855 after addition of 1 mL zearalenone (ZEN) solution (c = 5 µg/mL) to 50 mL fungal culture in vegetative (veg.) and generative (gen.) growth phase and subsequent ZEN incubation over a period of 10 to 12 days ; analyses were conducted in triplicate; a response factor for ZEN14S/ZEN of 11 and for α-ZEL-S/ZEN of 2.3 was applied; bars represent mean values ± SEM (standard error of the mean).


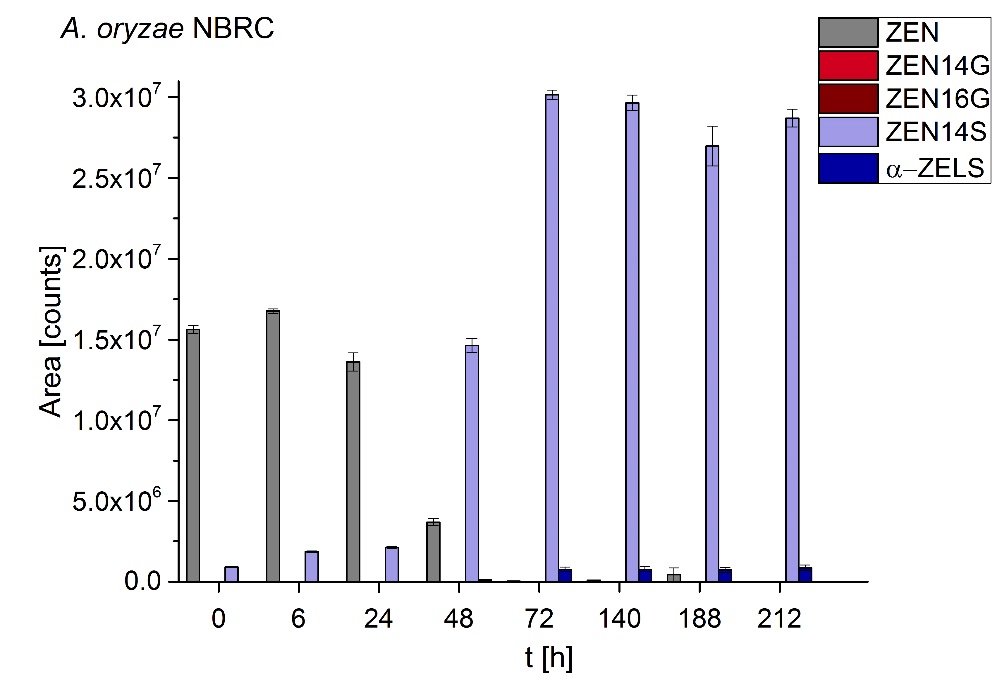


**Figure S2.** Formation of ZEN-14-sulfate (ZEN14S), ZEN-14-glucoside (ZEN14G), ZEN-16-glucoside (ZEN16G) and α-ZEL-sulfate (α-ZELS) by *Aspergillus oryzae* NBRC 100959 after addition of zearalenone (ZEN) contaminated rice flour (containing 4 mg ZEN) to 50 mL potato dextrose liquid media and subsequent fungal incubation over a period of 9 days; analyses were conducted in triplicate; a response factor for ZEN14S/ZEN of 11 was applied; bars represent mean values ± SEM (standard error of the mean).


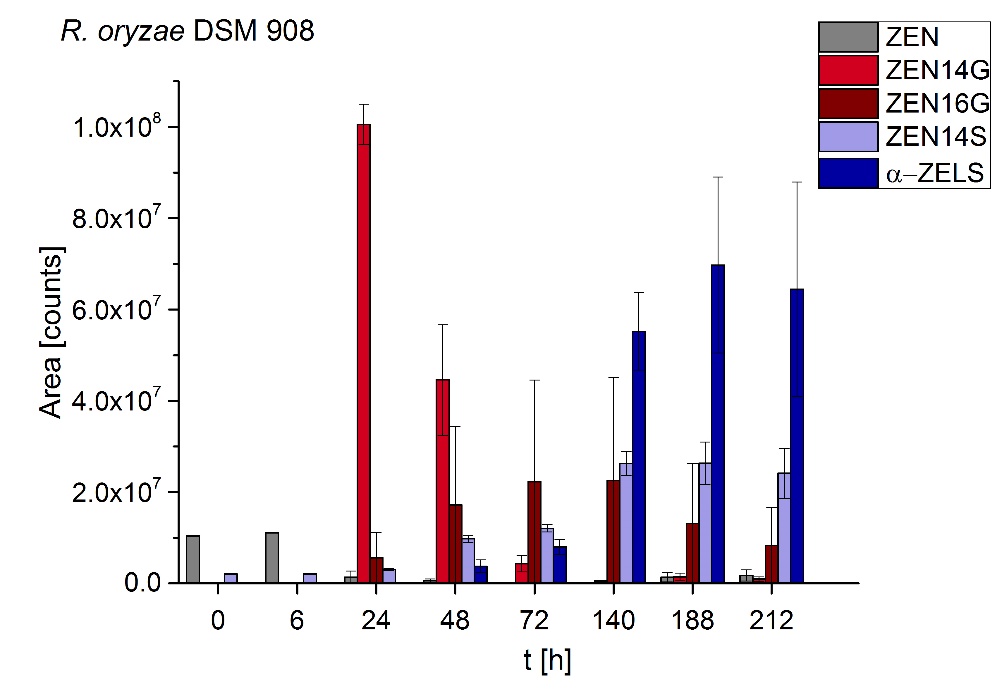


**Figure S3.** Formation of ZEN-14-sulfate (ZEN14S), ZEN-14-glucoside (ZEN14G), ZEN-16-glucoside (ZEN16G) and α-ZEL-sulfate (α-ZELS) by *Rhizopus oryzae* DSM 908 after addition of zearalenone (ZEN) contaminated rice flour (containing 4 mg ZEN) to 50 mL potato dextrose liquid media and subsequent fungal incubation over a period of 9 days; analyses were conducted in triplicate; a response factor for ZEN14S/ZEN of 11 was applied; bars represent mean values ± SEM (standard error of the mean).


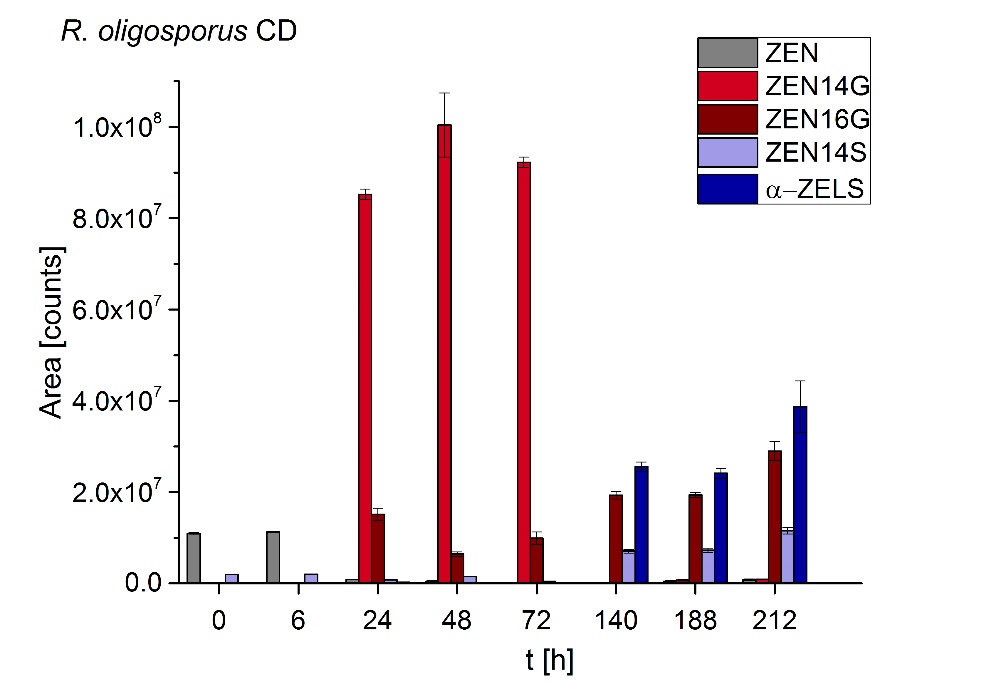


**Figure S4.** Formation of ZEN-14-sulfate (ZEN14S), ZEN-14-glucoside (ZEN14G), ZEN-16-glucoside (ZEN16G) and α-ZEL-sulfate (α-ZELS) by *Rhizopus oligosporus* CD after addition of zearalenone (ZEN) contaminated rice flour (containing 4 mg ZEN) to 50 mL potato dextrose liquid media and subsequent fungal incubation over a period of 9 days; analyses were conducted in triplicate; a response factor for ZEN14S/ZEN of 11 was applied; bars represent mean values ± SEM (standard error of the mean).

**Figure S5.** ^1^H-qNMR spectrum of ZEN-14-G in DMSO-d6; standard: trimesic acid trimethyl ester.


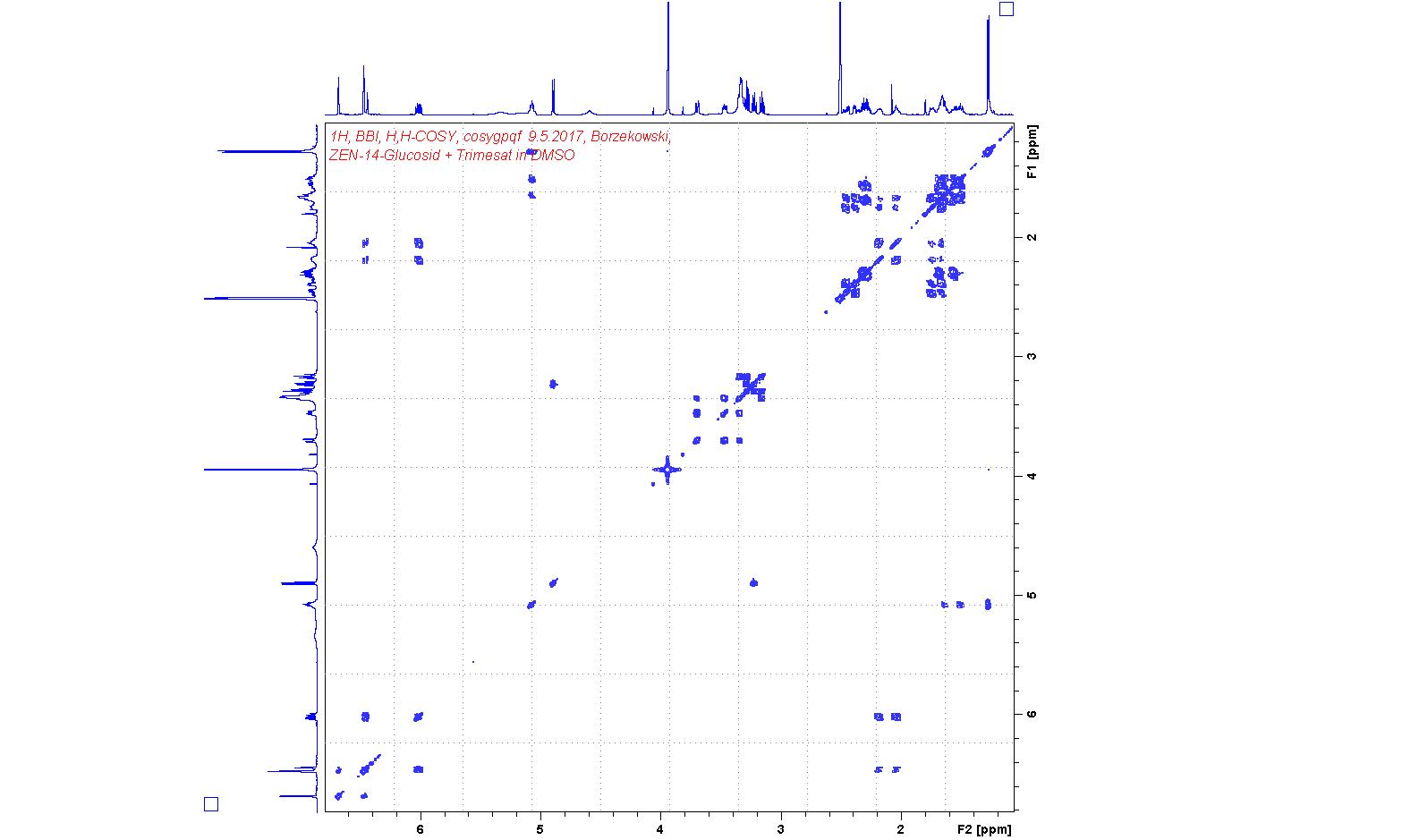


**Figure S6.** HH-COSY spectrum of ZEN-14-G.


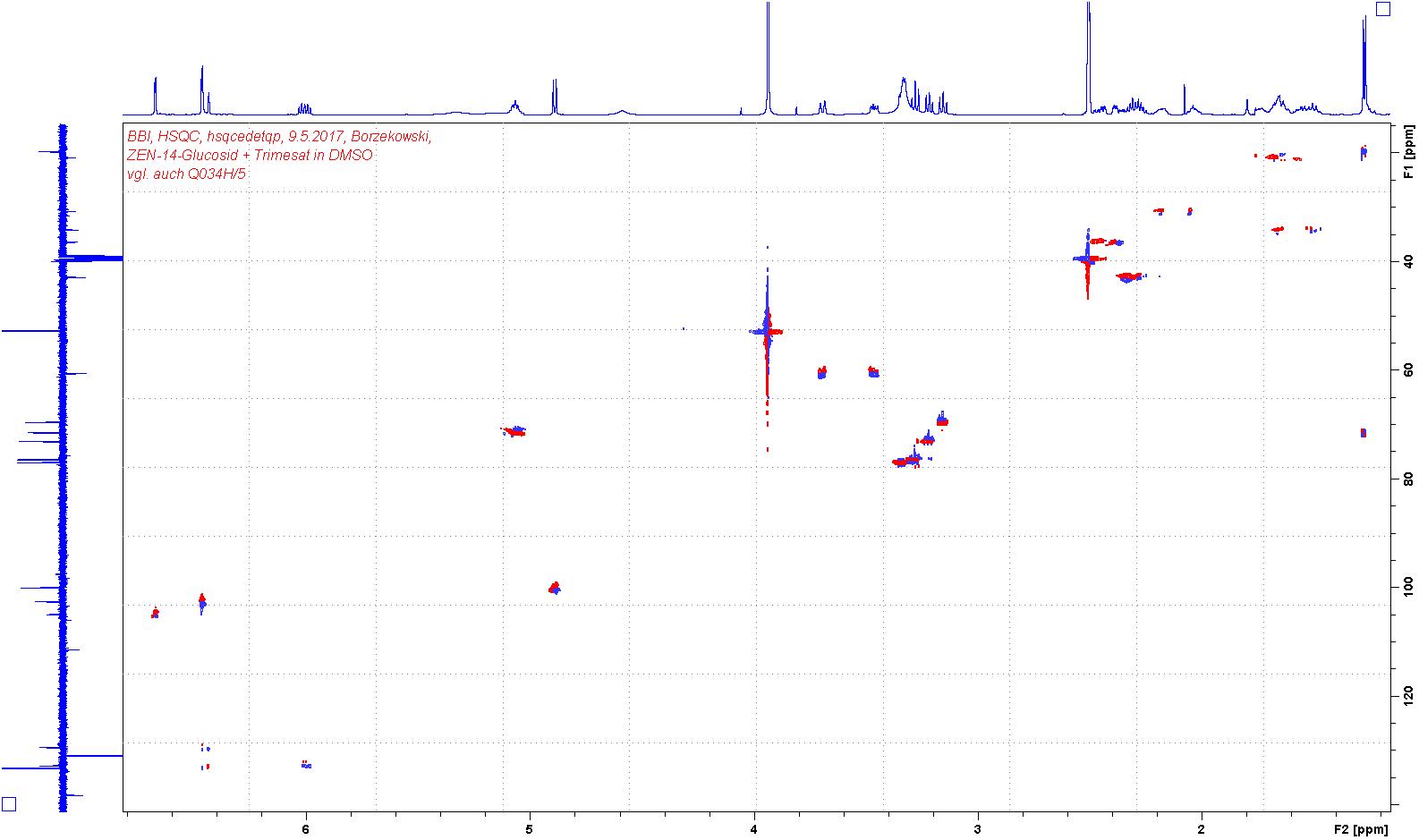


**Figure S7.** HC-HSQC spectrum of ZEN-14-G.


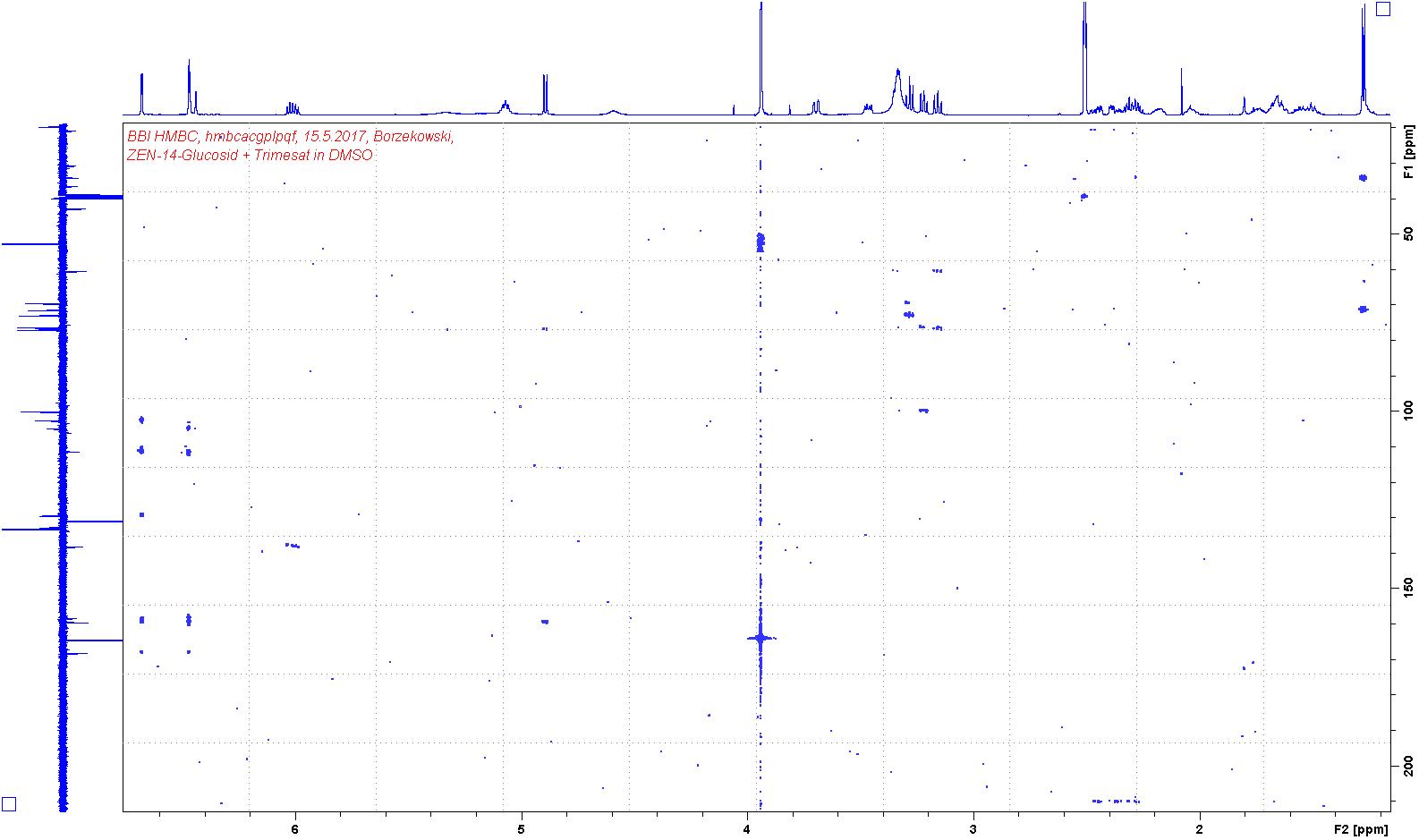


**Figure S8.** HC-HMBC spectrum of ZEN-14-G.

**Figure S9.** ^1^H-qNMR spectrum of ZEN-14-S in DMSO-d6; standard: trimesic acid trimethyl ester.


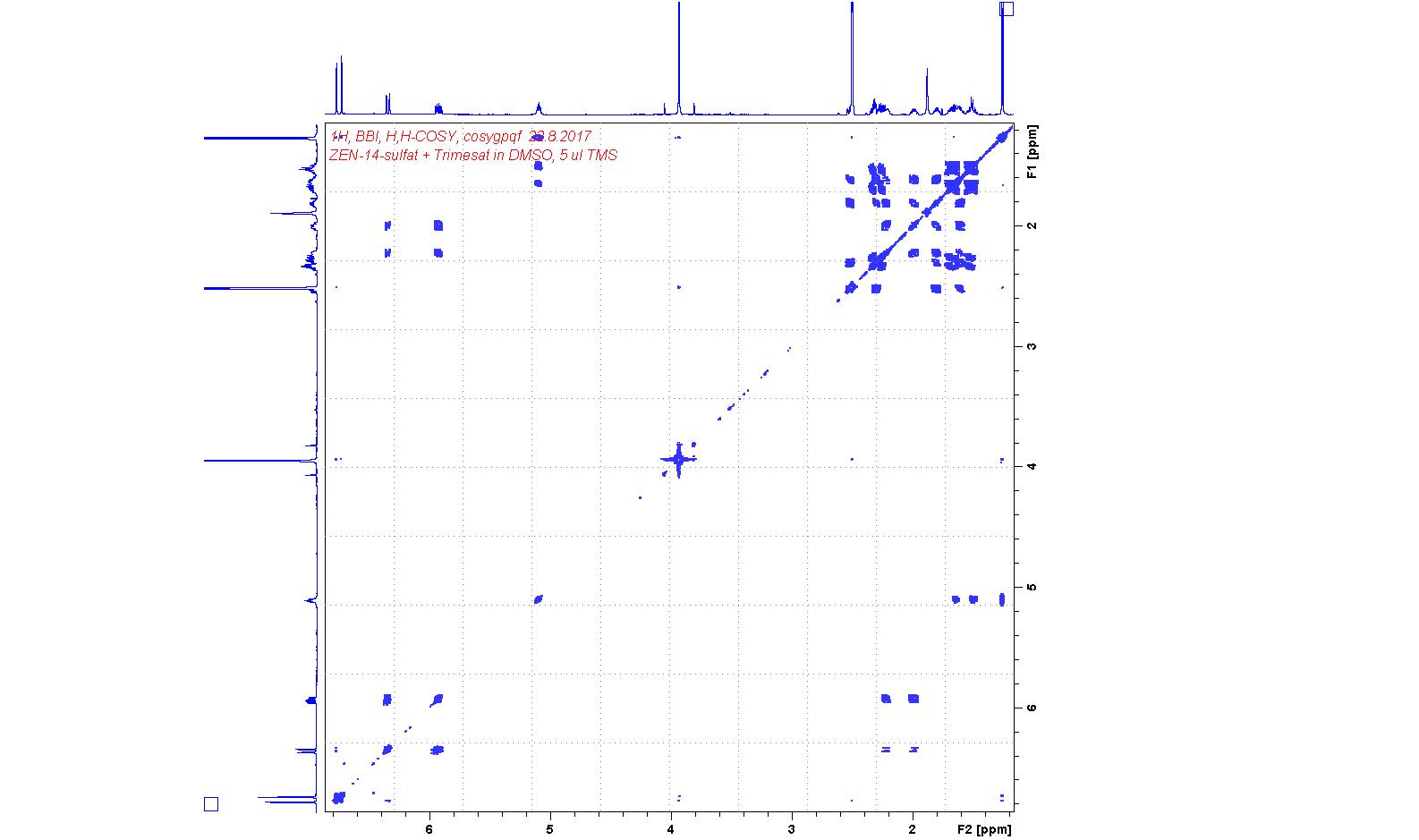


**Figure S10.** HH-COSY spectrum of ZEN-14-S.

**
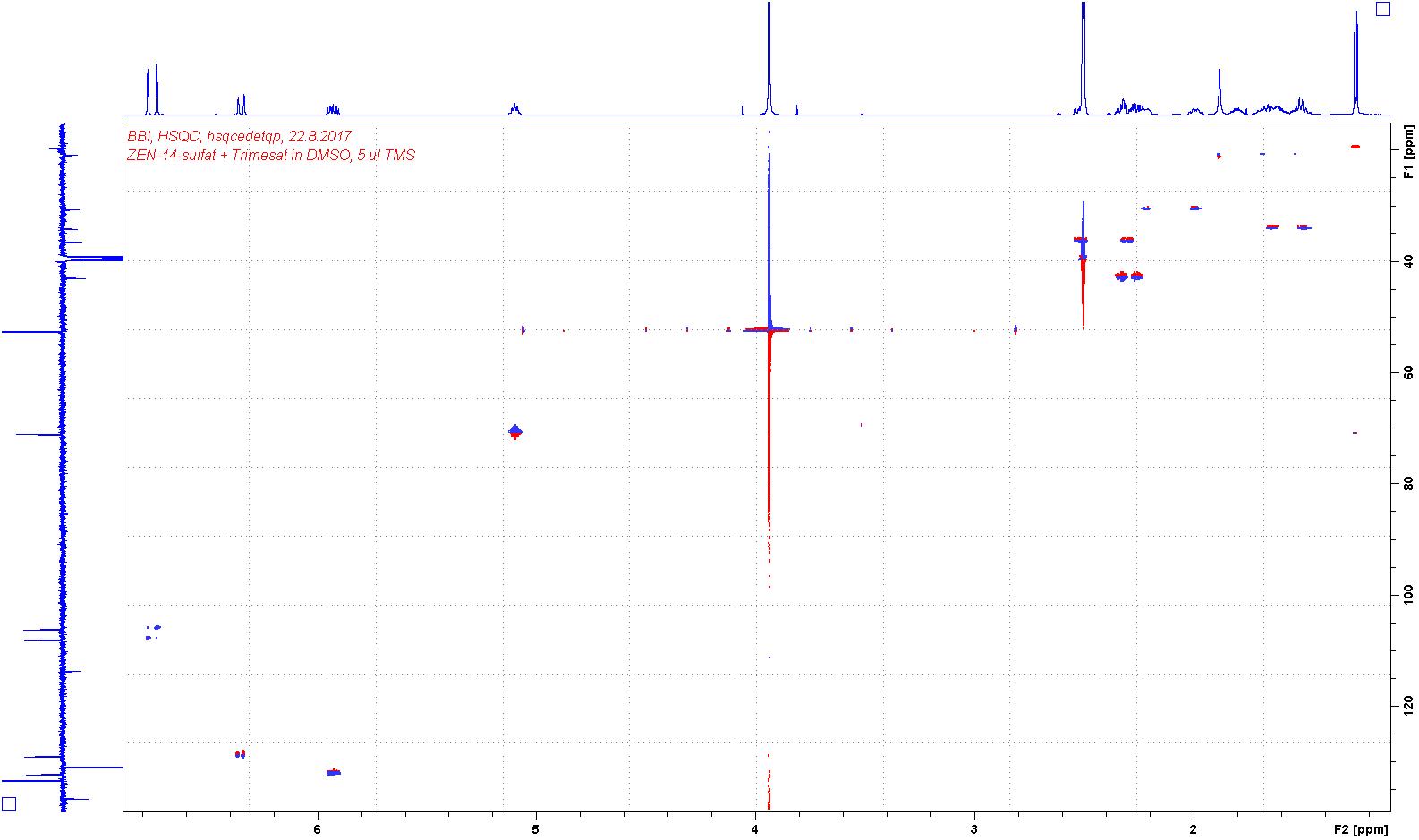
**

**Figure S11.** HC-HSQC spectrum of ZEN-14-S.


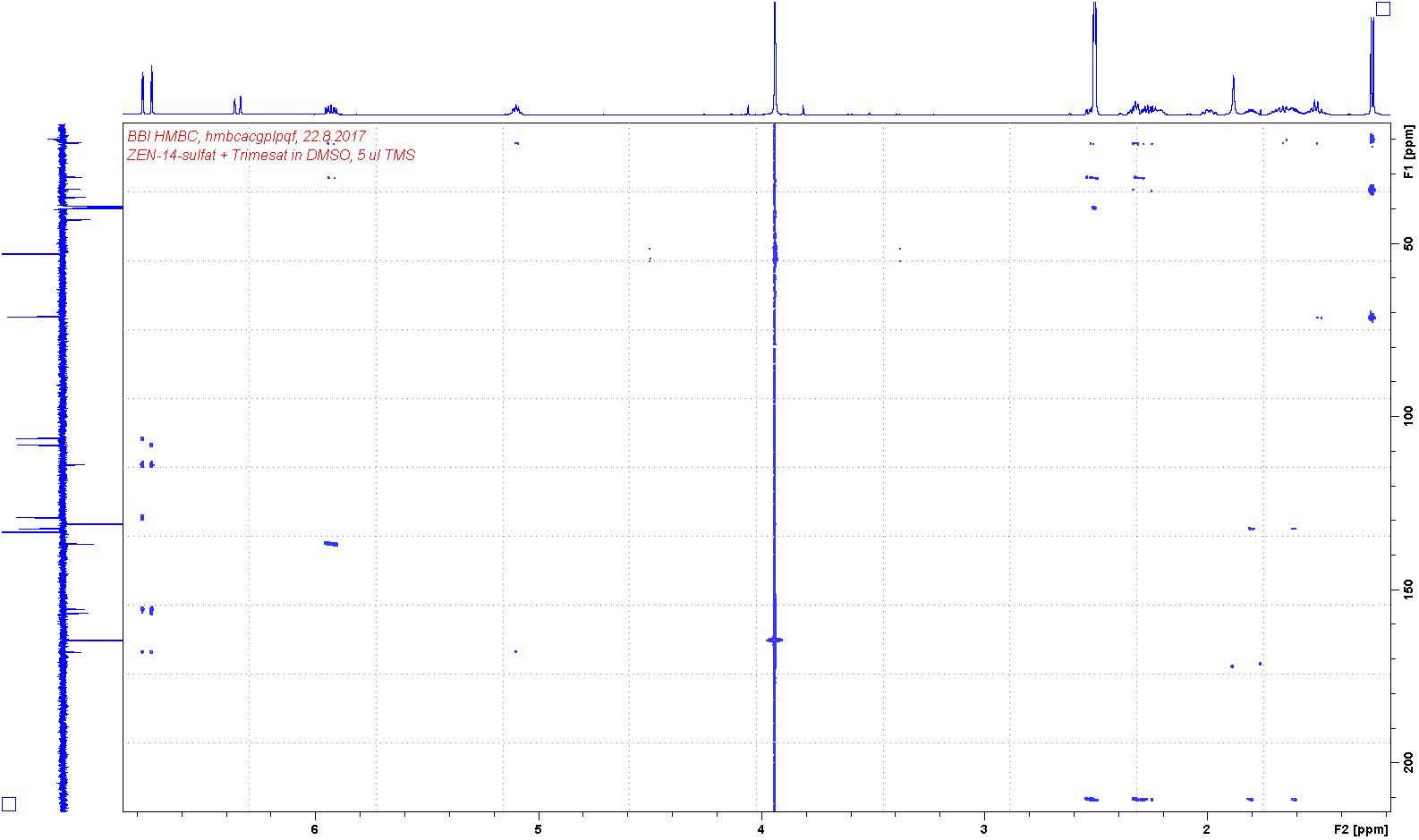


**Figure S12.** HC-HMBC spectrum of ZEN-14-S.

**Figure S13.** ^1^H-qNMR spectrum of ZEN-16-G in DMSO-d6; standard: trimesic acid trimethyl ester.


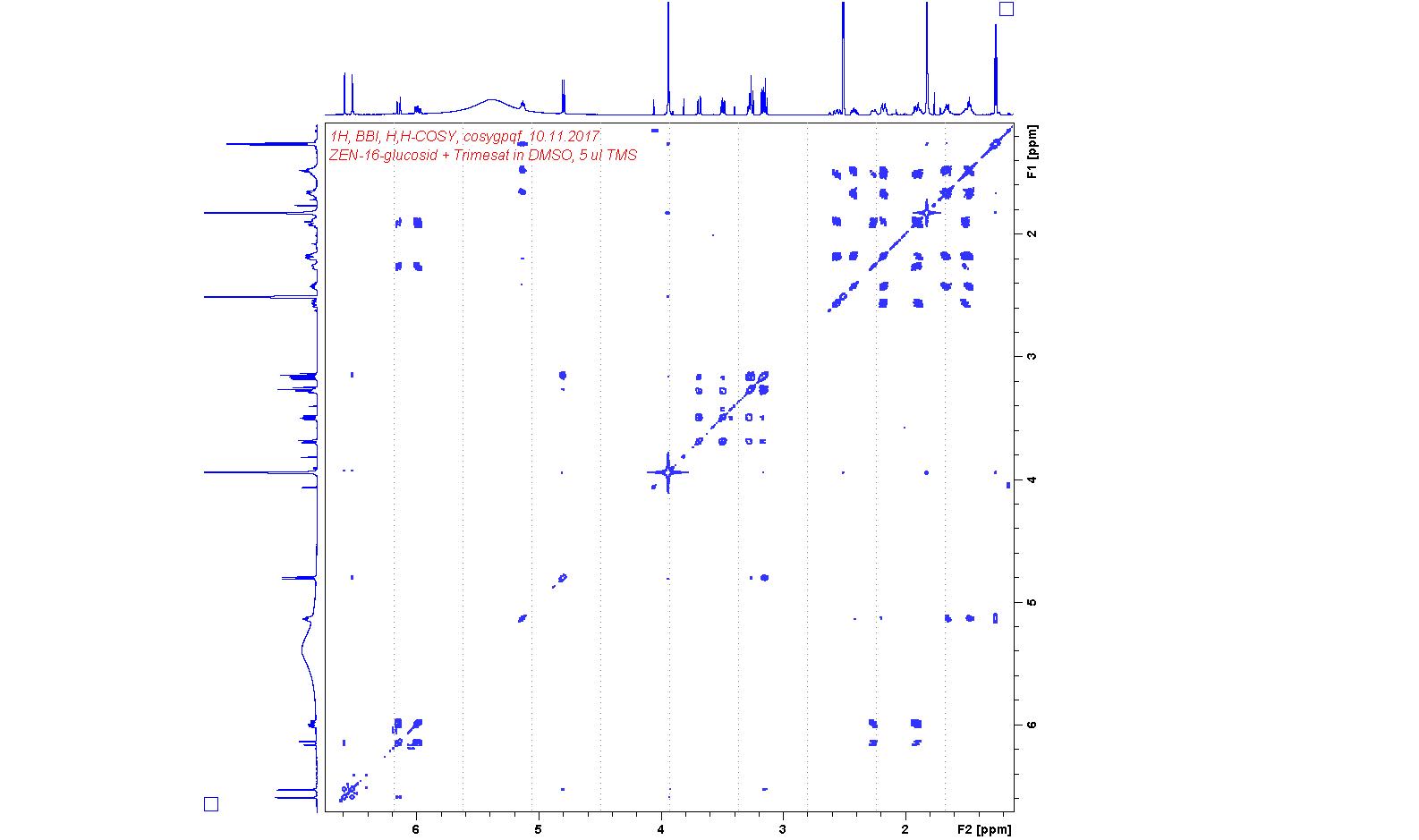


**Figure S14.** HH-COSY spectrum of ZEN-16-G.

**
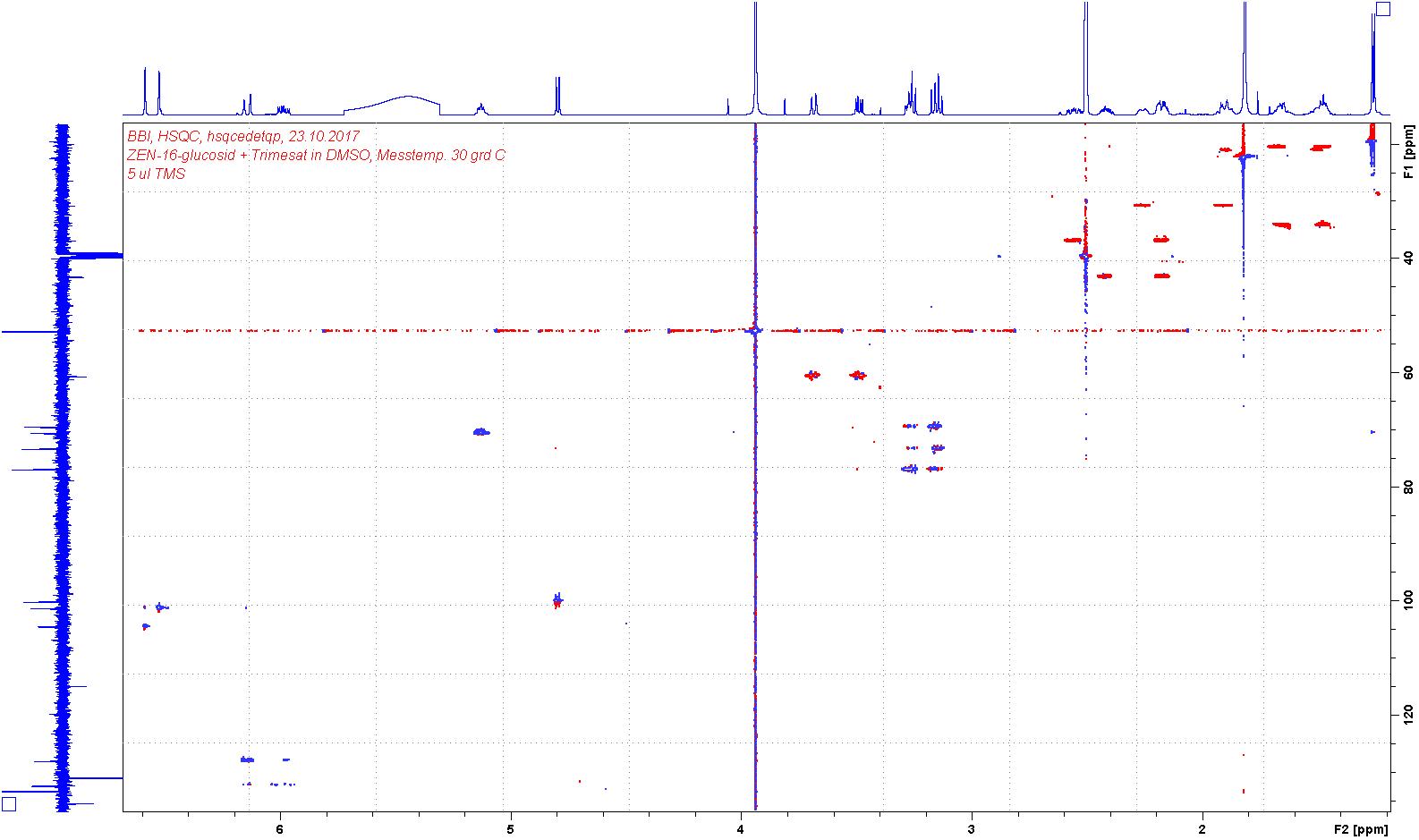
**

**Figure S15.** HC-HSQC spectrum of ZEN-16-G.

**
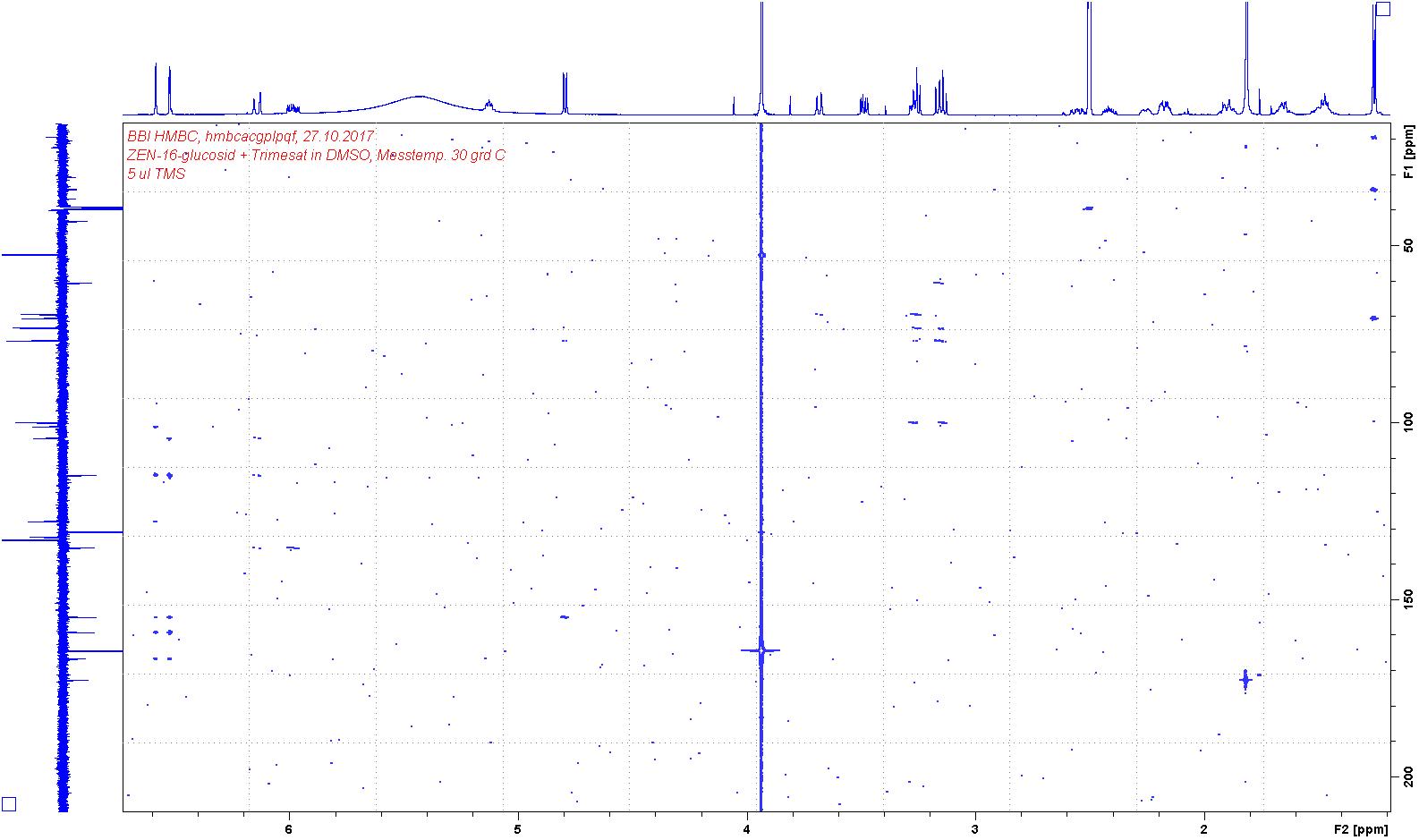
**

**Figure S16.** HC-HMBC spectrum of ZEN-16-G.

**Figure S17.** Comparison of the ^1^H-NMR spectra of the biosynthesized ZEN-14-S (above) and the chemically synthesized ZEN-14-S standard (below).
